# Loss of Atp8a2 drives neurodegeneration through the dysregulation of spatiotemporal phosphatidylserine externalization in mature neurons

**DOI:** 10.1038/s41419-026-09097-y

**Published:** 2026-07-22

**Authors:** Adriana Schneider, Alexandra B. Merkel, Nunzio Perta, Lisa Ruff, Netta Ussyshkin, Paula Zimmer, Bahar Aksan, Amandine Lepeuve, Laura D’Andrea, Silvia Pelucchi, Elena Marcello, Daniele Di Marino, Daniela Mauceri

**Affiliations:** 1 Department of Neurobiology, Interdisciplinary Centre for Neurosciences (IZN), Heidelberg University, Heidelberg, Germany; 2 Department of Life and Environmental Sciences, Marche Polytechnic University, Ancona, Italy; 3 Department of Neuroscience, Istituto di Ricerche Farmacologiche Mario Negri IRCCS, Milan, Italy; 4 Dept. Molecular and Cellular Neuroscience, Institute of Anatomy and Cell Biology, Marburg University, Marburg, Germany; 5 Department of Pharmacological and Biomolecular Sciences “Rodolfo Paoletti”, Università degli Studi di Milano, Milan, Italy

**Keywords:** Cell death in the nervous system, Molecular neuroscience

## Abstract

Phosphatidylserine (PS) asymmetry in plasma membranes is critical for cellular functions and serves as an apoptotic signal in many cell types. However, in mature neurons, the molecular mechanisms governing PS distribution, its precise regulation, and its functional significance beyond apoptosis and development remain poorly understood — particularly in the context of neurodegeneration. Here, we mapped the spatiotemporal dynamics of PS exposure in mature hippocampal neurons under physiological and pathological conditions using time-lapse imaging, revealing specific PS externalization hotspots at dendritic branching points. Using multiple in vitro and in vivo neurodegeneration models combined with molecular modeling, RNA interference, pharmacological interventions, and biochemical assays, we identified Atp8a2 as the primary regulator of PS asymmetry in mature neurons beyond its known roles in development. Notably, Atp8a2 expression levels — rather than its flippase activity alone — were essential for maintaining neuronal structural integrity and viability. Atp8a2 expression was significantly altered by neurotoxic stimuli and in multiple mouse models of neurodegeneration. Reduced Atp8a2 expression led to increased PS exposure, compromised neuronal architecture, and heightened susceptibility to degeneration, whereas Atp8a2 overexpression conferred substantial neuroprotection. The distinction between Atp8a2’s enzymatic activity and expression level reveals a mechanism of neuronal homeostasis linking PS regulation to structural integrity and survival, possibly through association with cytoskeletal protein networks. Thus, Atp8a2 expression is a critical determinant of mature neuronal viability, presenting a potential target for neuroprotective strategies in neurodegeneration.

## Introduction

The plasma membrane of eukaryotic cells is a highly specialized structure, defined not just by its fundamental role as a barrier but also by the striking asymmetry in its lipid distribution [1]. This asymmetry is characterized by the distinct distribution of specific phospholipids: phosphatidylserine (PS), phosphatidylinositol (PI), and phosphatidylethanolamine (PE) are predominantly localized to the inner leaflet, while glycolipids, phosphatidylcholine (PC), and sphingomyelin are confined to the outer leaflet. While the intrinsic chemical properties of these phospholipids restrict their passive translocation across the membrane, cells have evolved dynamic molecular machinery to regulate phospholipid distribution in response to various physiological and pathological signals.

PS translocation to the cell surface traditionally signals apoptosis; however, recent evidence reveals its occurrence in various non-apoptotic and physiological contexts, suggesting broader functional roles in cellular signaling and tissue homeostasis [2, 3]. This expanded understanding of PS dynamics raises important questions about its regulation and function in specialized and highly polarized cell types, particularly neurons, where membrane composition is crucial [4, 5].

In neurons, PS externalization presents a particularly complex and unresolved picture. While PS exposure marks apoptosis in many cell types, neuronal PS externalization has been observed under non-apoptotic, degenerative conditions [6, 7]. Accumulating evidence further indicates that PS exposure in neurons can be transient and reversible, occurring in stressed yet viable cells [8]. This transient PS display triggers microglia recognition, potentially initiating phagocytic responses that may contribute to synaptic pruning or neuronal loss [8]. Such interactions highlight a delicate balance that may influence neuroinflammatory processes and neurodegenerative disease pathogenesis.

Three types of enzymes regulate PS asymmetry: floppases, which mediate outward PS translocation; flippases, which catalyze the ATP-dependent transport of PS to the inner leaflet; and scramblases, which enable bidirectional lipid movement. While these enzymes comprise nearly 20 different proteins, the specific molecular determinants controlling PS asymmetry in mature neurons and their role in neurodegeneration remain unclear.

Here, we reveal that PS externalization in mature neurons follows distinct spatiotemporal patterns, concentrating at specific structural domains. We demonstrate that non-apoptotic neurotoxic conditions trigger characteristic PS exposure signatures and dramatically alter the expression of Atp8a2 flippase. We establish a role for Atp8a2 in mature neurons that extends beyond its previously reported developmental roles, revealing a dual role in maintaining both PS asymmetry and structural integrity. Further, this protective function depends on Atp8a2 expression levels rather than its flippase activity alone, uncoupling enzymatic function from neuronal integrity and survival. Our findings determine Atp8a2 expression levels as a key factor in neuronal vulnerability to damage and identify a potential therapeutic target for neurodegenerative disorders.

## Results

### Excitotoxicity triggers spatially organized phosphatidylserine externalization in mature neurons

**Fig. 1:**
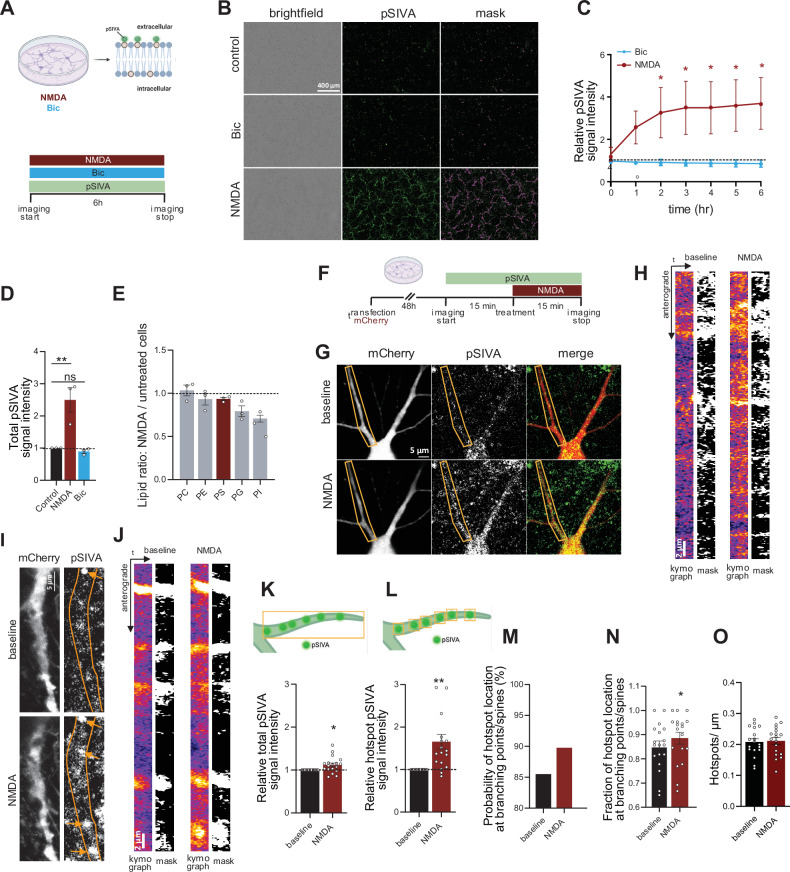
Neuronal PS externalization is increased and spatially organized following excitotoxicity. **A**–**D** Time-lapse analyses of PS exposure in hippocampal neurons upon induced synaptic activity (Bic) or excitotoxicity (NMDA). **A** Schema of pSIVA time-lapse imaging experiments. Icons created in BioRender. Mauceri, D. (2026) https://BioRender.com/tl3ez3u. **B** Representative pSIVA images of neurons treated with NMDA, Bic, or untreated 2 h after treatments. Images acquired with Incucyte® S3 Live-Cell Analysis System. Scale bar = 400 µm. Relative pSIVA fluorescence intensity over time (**C**) and integrated (**D**), in neurons treated with NMDA, Bic, or untreated and normalized to initial timepoint and untreated control. *N* = 4 independent cultures. Two-way ANOVA with repeated measures with Dunnett’s post hoc test (**C**), or one-way ANOVA with Dunnett’s post hoc test (**D**). **E** Mass spectrometry analyses of phospholipid levels in hippocampal neurons with NMDA treatment, normalized to untreated controls. Graph shows relative abundances for phospholipid classes. *N* = 4 independent cultures. One sample *t*-tests. **F**–**O** Live imaging analyses of dendritic PS exposure ± NMDA treatment. **F** Schema of pSIVA live imaging in mCherry-transfected hippocampal neurons ± NMDA treatment. Icon created in BioRender. Mauceri, D. (2026) https://BioRender.com/qbpkcnn. **G** Representative proximal dendrites of mCherry-transfected neurons with pSIVA signal at baseline and 10 min post-NMDA. Analyzed dendrite in yellow. Scale bar = 5 µm. **H** Representative kymographs and binary masks of pSIVA signal from (**G**) during 15 min baseline and 15 min post-NMDA. Scale bar = 2 µm. Representative images (**I**) and corresponding kymographs (**J**) of pSIVA live imaging in higher-magnification of an mCherry-transfected proximal dendrite of a hippocampal neuron, 10 min after ± NMDA treatment. Dendritic region within pSIVA signal is highlighted in yellow. Scale bars: images = 5 µm; kymographs = 2 µm. Relative pSIVA fluorescence intensity of (**K**) total dendrite area or (**L**) within hotspots, normalized to baseline. Icons created in BioRender. Mauceri, D. (2026) https://BioRender.com/x032fp9. **M**–**O** Analysis of pSIVA hotspots pattern. **M** Probability of hotspots at dendritic branching points/spines relative to baseline (mixed logistic regression). **N** Fraction of hotspots at dendritic branching points / spines over total hotspots. **O** Hotspots density along dendrites. *N* = 4 independent cultures, 18 neurons, 1-2 dendrites per neuron. One-sample *t*-test (**K**, **L**), or two-tailed paired *t*-test (**N**, **O**). Graphs display mean ± SEM. Single values are represented as data points. ^**^p < 0.01; ^*^p < 0.05; ns non-significant *p* > 0.05.

To investigate the dynamic mechanisms governing PS localization in mature neurons, we employed time-lapse imaging with pSIVA (Polarity Sensitive Indicator of Viability & Apoptosis; Fig. 1A) [7, 9]. pSIVA is a thiol-reactive fluorescent probe, derived from Annexin and featuring IANBD, suitable for the detailed real-time spatio-temporal analysis of irreversible and reversible PS surface exposure [7, 9]. pSIVA specifically detects PS externalization: when it binds to PS on the surface of cells, this results in a conformational change and the production of a fluorescent signal, and if PS is flipped back into the intracellular leaflet of the membrane, pSIVA is released and the fluorescence stops [7, 9].

We exposed hippocampal neurons to two different stimuli: bicuculline (Bic) to enhance synaptic activity or NMDA to induce excitotoxicity and neuronal death characteristic of multiple pathological conditions and distinct from apoptosis. Bic promotes action potential bursting and induces robust calcium rises [10], whereas NMDA, during bath application, facilitates calcium entry through synaptic as well as extrasynaptic NMDA receptors (eNMDARs), resulting in toxicity mechanisms relevant to several acute and chronic neurodegenerations [11–13]. Our experiments revealed that, compared to untreated cultures or Bic treatment, NMDA application caused a robust and sustained increase of pSIVA signal (Fig. 1B–D). We obtained similar results with a different imaging system, excluding possible technical artifacts (Supplementary Fig. 1A–C). As extracellular vesicles (EV) are known to carry PS on their outer leaflet [14], we confirmed that pharmacological inhibition of EV biogenesis did not affect the NMDA-induced pSIVA signal increase, supporting the specificity of the pSIVA readout for neuronal PS externalization (Supplementary Fig. 1A, D). Next, to further confirm our observation that a non-apoptotic signal — the toxic activation of eNMDARs — would indeed trigger PS externalization, we used two additional strategies: glutamate exposure and dl-threo-β-benzyloxyaspartic acid (DL-TBOA) treatment (Supplementary Fig. 1A, E, F). DL-TBOA inhibits glutamate up-take systems, increasing glutamate levels in the extrasynaptic space and stimulating eNMDARs, ultimately causing toxicity [15]. Both treatments resulted in PS exposure similar to NMDA treatment (Supplementary Fig. 1A, E, F). Next, we tested whether the detected increase in PS exposure was due to differences in total PS levels. Mass spectrometry-based lipidomics confirmed that the observed changes reflected PS redistribution towards the outer leaflet rather than alterations in total PS levels (Fig. 1E). Together, these findings demonstrate that excitotoxicity triggers a robust externalization of PS on the neuronal surface, prompting us to ask whether this exposure follows a spatially organized pattern.

Thus, to characterize the spatial dynamics of PS externalization during neurotoxicity, we monitored pSIVA on the proximal dendrites of hippocampal neurons during baseline and after NMDA application (Fig. 1F). Dendritic morphology was visualized via mCherry expression (Fig. 1G, I). Specific subcellular events such as membrane blebbing were not assessed in this analysis. In agreement with our previous observations (Fig. 1A–D), following NMDA application, pSIVA fluorescence intensity increased significantly along dendrites (Fig. 1G–K). Notably, the pSIVA signal was not uniformly distributed but clustered into distinct “hotspots” with minimal diffused signal (Fig. 1H, J). After NMDA treatment, these hotspots showed significantly increased fluorescence intensity (Fig. 1H, J, L), with the effect being stronger than the observed global rise in PS externalization (Fig. 1G–K). Closer inspection of these hotspots revealed a distinct pattern: under baseline conditions, they frequently localized at dendritic branching points and spines (Fig. 1M, N). While NMDA treatment did not increase the overall density of hotspots (Fig. 1O), it significantly enhanced their localization at these structural sites (Fig. 1M, N). This suggests that excitotoxicity redistributes existing PS exposure hotspots toward key dendritic locations rather than increasing their overall number.

Taken together, these findings reveal that PS externalization in mature neurons occurs in a highly organized spatial pattern, with preferential exposure at key structural points.

### Neurodegenerative insults selectively downregulate Atp8a2 across multiple disease models

**Fig. 2:**
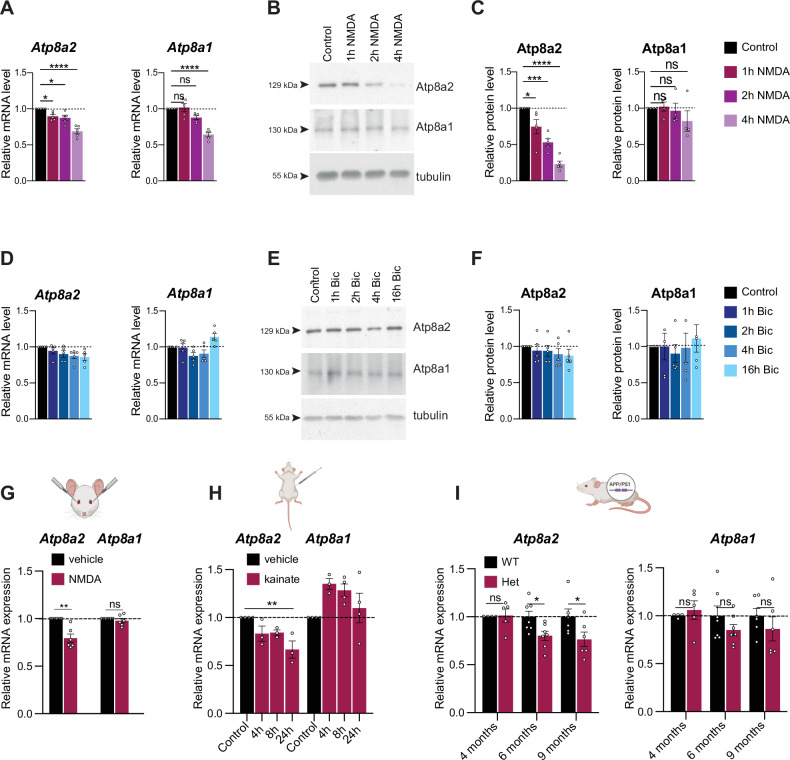
Atp8a2 expression is affected by excitotoxicity and downregulated in neurodegenerative conditions. **A** QRT-PCR analysis of *Atp8a1* and *Atp8a2* mRNA expression in hippocampal neurons ± NMDA treatment for 1, 2 or 4 h. Expression levels were normalized to *Gusb* and control. *N* = 5 independent cultures. **B**, **C** Immunoblot analyses of Atp8a2 and Atp8a1 protein expression in hippocampal neurons ± NMDA treatment for 1, 2 or 4 h. **B** Representative immunoblots of Atp8a2, Atp8a1, and tubulin. **C** Atp8a2 or Atp8a1 expression levels normalized to tubulin and control. *N* = 4-5 independent cultures. **D** QRT-PCR analysis of mRNA expression of *Atp8a2* and *Atp8a1* in hippocampal neurons ± Bic treatment for 1, 2, 4 or 16 h. Expression levels were normalized to *Gusb* and control. *N* = 5 independent cultures. **E**, **F** Immunoblot analyses of Atp8a2 and Atp8a1 protein expression in hippocampal neurons ± Bic treatment for 1, 2, 4 or 16 h. **E** Representative immunoblots of Atp8a2, Atp8a1, and tubulin. **F** Atp8a2 or Atp8a1 expression levels normalized to tubulin and control. *N* = 4-5 independent cultures. One-way ANOVA with Dunnett’s post hoc test (**A**, **C**, **D**, **F**). **G** Wildtype (WT) mice were intravitreally injected with NMDA or vehicle. QRT-PCR analysis of *Atp8a1* and *Atp8a2* mRNA expression in the retina 24 h after intravitreal injection. *N* = 6 mice. One sample *t*-test. Icon created in BioRender. Mauceri, D. (2026) https://BioRender.com/veis2x4. **H** WT mice were intraperitoneally injected with kainate or vehicle. QRT-PCR analysis of mRNA expression of *Atp8a1* and *Atp8a2* in the hippocampus 4, 8 or 24 h after injection. *N* = 3-4 mice. One-way ANOVA with Dunnett’s post hoc test. Icon created in BioRender. Mauceri, D. (2026) https://BioRender.com/3850dqm. **I** Hippocampi of WT mice and APP/PS1 mice of 4, 6, and 9 months of age were analyzed by QRT-PCR analysis for mRNA expression of *Atp8a1* and *Atp8a2*. *N* = 5-6 mice. Multiple unpaired t-test. Icon created in BioRender. Mauceri, D. (2026) https://BioRender.com/9du2bci. Expression levels were normalized to *Gusb* (**H**, **I**) or *Gapdh* (**G**) and controls. Graphs represent mean ± SEM. Single values are represented as data points. ^****^p < 0.0001; ^***^p < 0.001; ^**^p < 0.01; ^*^p < 0.05; ns non-significant *p* > 0.05.

We hypothesized that the observed PS externalization (Fig. 1, and Supplementary Fig. 1) could result from toxic dysregulation of enzymes governing PS shuttling. The specific enzymes responsible for PS dynamics in mature neurons have not been fully characterized. Based on literature and public data repositories, we examined the expression of 13 confirmed and putative candidates and interaction partners responsible for shuttling PS in neurons (Fig. 2A, and Supplementary Fig. 2A): flippases (*Atp8a1*, *Atp8a2*, *Atp11a*, *Atp11c*, *Atp8b2* [16, 17]), floppases (*Abca1* [18]), scramblases (*Plscr1*, *Plscr3*, *Plscr4*, *Xkr4*, *Xkr8*, *Tmem16f* [19]) and the accessory ß-subunit *Cdc50a* [20]. *cFos* was used as positive control (Supplementary Fig. 2A). NMDA treatment significantly decreased mRNA levels of the flippases *Atp8a1* and *Atp8a2* while all the other tested genes remained unaffected (Fig. 2A, and Supplementary Fig. 2A). At the protein level, only Atp8a2 showed a significant NMDA-dependent reduction, while Atp8a1 remained stable (Fig. 2B, C), suggesting that additional mechanisms may regulate its expression. In contrast, enhanced synaptic activity via Bic treatment, which did not affect PS exposure (Fig. 1A–D, and Supplementary Fig. 1A–C), left both mRNA and protein levels of Atp8a1 and Atp8a2 unaltered (Fig. 2D–F).

Elevated extracellular glutamate concentrations or NMDA receptor toxic activation have been linked to both acute and progressive neurodegenerative conditions [11, 12, 21, 22]. To determine whether Atp8a2 downregulation may represent a common neurodegenerative mechanism in vivo, we examined several mouse models of neurodegeneration. As Atp8a2 is expressed in multiple areas of the central nervous system [23–26], we used models targeting different regions. In retinal ganglion cells (RGCs), which are particularly vulnerable to excitotoxicity [27–30], a single intravitreal NMDA injection significantly reduced *Atp8a2* but not *Atp8a1* mRNA levels (Fig. 2G). Similarly, kainic acid-induced seizures in adult mice, typically triggering NMDAR-mediated excitotoxic cell death of CA1 pyramidal neurons [31, 32], caused a time-dependent decline in hippocampal *Atp8a2* expression while sparing *Atp8a1* (Fig. 2H). Dysregulated glutamate signaling has also been implicated in chronic neurodegenerative conditions such as Alzheimer’s Disease (AD) [12]. We analyzed *Atp8a2* and *Atp8a1* in the hippocampus of a transgenic mouse model of AD at different stages: 4 months represents the asymptomatic stage with no clear impairment in cognitive performance, 6 months is an initial symptomatic stage with first cognitive defects, and 9 months is a full-blown pathology state accompanied by cognitive impairment and severe amyloid deposition [33–35]. *Atp8a2* levels, but not *Atp8a1*, were significantly reduced in the hippocampus of heterozygous (HET) mice at 6 and 9 months compared to wildtype (WT) controls (Fig. 2I).

These findings establish a consistent pattern across multiple neurodegenerative conditions: selective downregulation of Atp8a2 expression, correlating with increased PS externalization and suggesting a convergent pathological mechanism across diverse neurodegenerative diseases.

### Atp8a2 specifically controls PS asymmetry in mature neurons

**Fig. 3:**
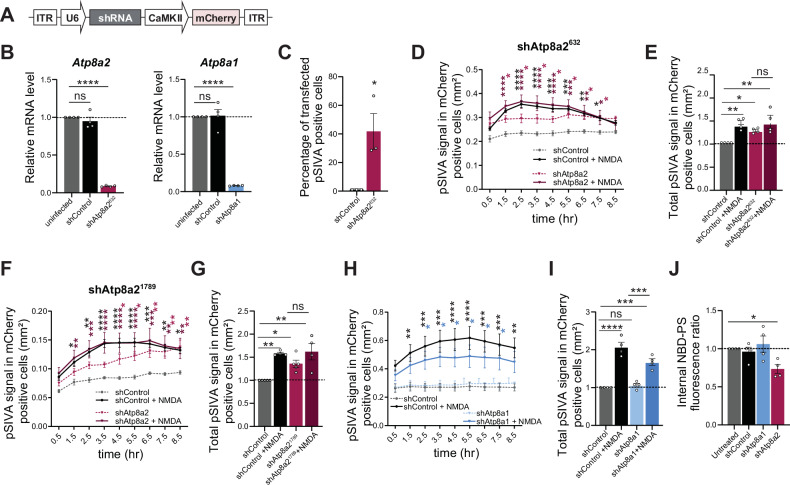
Reduced Atp8a2 expression elevates PS externalization. **A** Schema of the used constructs. **B** QRT-PCR analysis of *Atp8a2* and *Atp8a1* mRNA expression in hippocampal neurons infected as indicated, or uninfected. Expression normalized to *Gusb* and uninfected control. *N* = 4 independent cultures. One-way ANOVA with Dunnett’s post hoc test. **C** Percentage of pSIVA positive hippocampal neurons transfected with shAtp8a2 or shControl. *N* = 3 independent cultures. Two-tailed unpaired *t*-test. **D**–**I** Time-lapse analyses of PS exposure in shAtp8a2- or shAtp8a1-transfected neurons ± NMDA treatment. Relative pSIVA signal on mCherry-positive neurons (shAtp8a2^632^) transfected and treated, over time (**D**) and integrated (**E**) and normalized to the initial mCherry-positive area (**D**) and untreated shControl (**E**). Relative pSIVA signal on mCherry-positive neurons (shAtp8a2^1789^), over time (**F**) and integrated (**G**) and normalized to the initial mCherry-positive area (**F**) and untreated shControl (**G**). Relative pSIVA signal on mCherry-positive neurons (shAtp8a1), over time (**H**) and integrated (**I**) and normalized to the initial mCherry-positive area (**H**) and untreated shControl (**I**). *N* = 4 independent cultures. Two-way ANOVA with repeated measures with Dunnett’s post hoc test for over time analyses (**D**, **F**, **H**), or One-way ANOVA with Bonferroni’s post hoc test for integrated analyses (**E**, **G**, **I**). **J** Analyses of intracellular NBD-PS fluorescence in cultured mouse hippocampal neurons infected with shAtp8a2, shAtp8a1, shControl, or uninfected and normalized to uninfected control. *N* = 4 independent cultures. One-way ANOVA with Dunnett’s post hoc test. Graphs represent mean ± SEM. Single values are represented as data points. ^****^p < 0.0001; ^***^p < 0.001; ^**^p < 0.01; ^*^p < 0.05; ns non-significant *p* > 0.05.

Given our observations that toxic insults trigger both PS externalization (Fig. 1, and Supplementary Fig. 1) and reduced Atp8a2 expression (Fig. 2), we hypothesized that Atp8a2 plays a crucial role in preserving PS asymmetry in mature neurons. Since mutations or translocations affecting the human Atp8a2 gene lead to severe developmental deficits [23, 36–39], we employed RNA interference to knock down Atp8a2 expression in already mature neurons to evaluate its impact on PS asymmetry without affecting early neurodevelopmental stages and to model what may happen in case of an adult decline (Fig. 3A). We verified the efficiency of the generated shRNA construct (shAtp8a2^632^ [25]) in comparison to a scrambled control (shControl, Fig. 3B). Reducing Atp8a2 expression was sufficient to cause a significant increase in neurons displaying PS externalization at resting conditions (Fig. 3C). Next, we tested whether a reduction of Atp8a2 expression could alter the temporal dynamics of PS exposure. shAtp8a2^632^-transfected hippocampal neurons had, as expected, a significantly higher and constant exposure of PS (Fig. 3D, E). When challenged with NMDA, shAtp8a2^632^-transfected hippocampal neurons showed no additional increase in PS exposure, suggesting that Atp8a2 reduction had already maximized PS externalization (Fig. 3D, E). These findings were confirmed with a second, independent, already validated [40] shRNA targeting Atp8a2 (shAtp8a2^1789^ Fig. 3A, F, G). From here onwards we will refer to either construct as shAtp8a2.

In striking contrast, reducing Atp8a1 expression (Fig. 3A, B) had no effect on PS exposure, either at baseline or during excitotoxicity (Fig. 3H, I). Finally, we assessed the levels of PS present on the inner leaflet of cultured neurons. Fluorescently labeled PS (NBD-PS) gets rapidly incorporated in the plasma membrane and is subjected to similar distribution as endogenous PS. By selectively quenching the fluorescent signal from NBD-PS localized in the extracellular layer of the plasma membrane, the remaining signal reflects the amount of PS present in the intracellular leaflet [41]. Measurements of PS in the inner leaflet using NBD-PS confirmed that Atp8a2-deficient neurons had significantly reduced inner leaflet PS (Fig. 3J), consistent with increased PS externalization (Fig. 3C–G), while Atp8a1-deficient neurons showed no difference from controls. Taken together, these data indicate that Atp8a2 expression in mature neurons is a determining factor in the asymmetric distribution of PS in the inner leaflet.

**Fig. 4:**
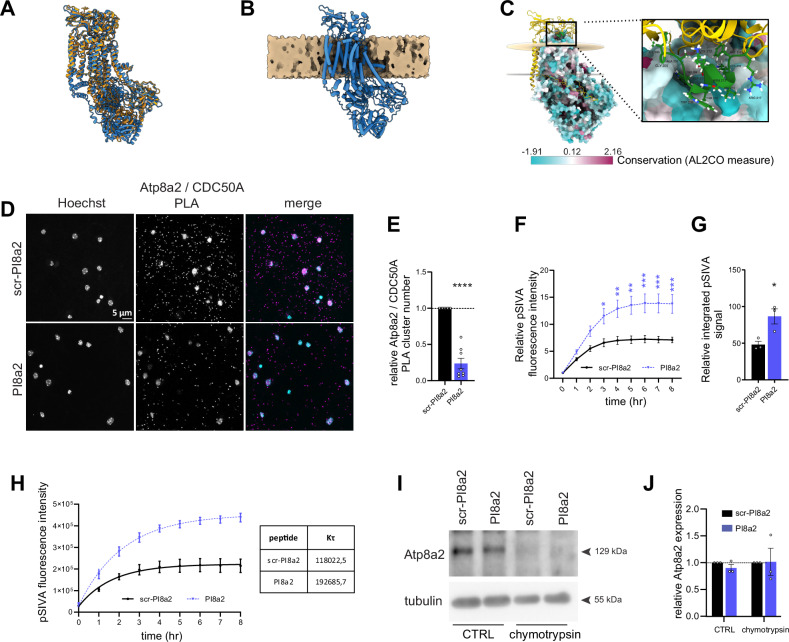
Neuronal Atp8a2 flippase activity is essential for maintaining PS asymmetry. **A**–**C** Structural modeling and conservation analysis of Atp8a2-CDC50A complex. **A** Predicted 3D structure of mouse Atp8a2-CDC50A complex (blue) generated using AlphaFold2-multimer, superimposed with human Atp8a1-CDC50A complex (orange; PDB ID: 6K7J). Superposition yields Cα RMSD of 1.27 Å, indicating high structural similarity. **B** Energy-minimized model of mouse Atp8a2-CDC50A complex in a lipid bilayer (tan slab). Atp8a2 and CDC50A shown as blue cylinders; cytosolic domains project below membrane, exocytoplasmic regions face upward. **C** Sequence conservation map of Atp8a2. Molecular surface colored by AL2CO entropy scores from multi-species eukaryotic flippases sequence alignment (cyan = variable; magenta = highly conserved). CDC50A is shown as a yellow ribbon. Inset highlights two short, low-conservation surface loops in Atp8a2 (residues 953–955 and 1009–1024) contacting CDC50A residues 205–217 (green), pinpointing region of CDC50A exploited for PI8a2 peptide design. **D**, **E** Proximity ligation assay (PLA) with antibodies against Atp8a2 and CDC50A in hippocampal neurons with scr-PI8A2 or PI8a2 treatment for 4 h. **D** Representative images of PLA signal in neurons treated as indicated. Nuclei were labeled with Hoechst. Scale bar = 5 µm. **E** PLA cluster number normalized on the number of Hoechst-labeled cells and on control. *N* = 8 images of 2 independent cultures. One sample *t*-test. **F**, **G** Time-lapse analyses of PS exposure in hippocampal neurons upon inhibition of Atp8a2 flippase activity by peptide treatment. Relative pSIVA fluorescence intensity over time (**F**) and integrated (**G**), in neurons treated with PI8a2 or scr-PI8a2, normalized to initial timepoint. *N* = 3 independent cultures. Two-way ANOVA with repeated measures with Bonferroni’s post hoc test for over time analysis (**F**), and two-tailed unpaired *t*-test for integrated analysis (**G**). **H** Non-linear regression analysis with one-phase association model, displaying Atp8a2 enzyme kinetics upon PI8a2 or scr-PI8a2 treatment. Data points represent mean pSIVA fluorescence intensity, plotted against time. Solid or dotted line indicates best-fit curve. Kτ-values (pSIVA fluorescence intensity/hr) obtained from the analysis determines rate of Atp8a2 inhibition. *N* = 3 independent cultures. **I**, **J** Immunoblot analysis of Atp8a2 plasma-membrane localization in hippocampal neurons after 4 h PI8a2 or scr-PI8a2 treatment, assessed by chymotrypsin proteolysis. **I** Representative immunoblots. **J** Atp8a2 levels normalized to tubulin and respective control. *N* = 3 independent cultures. One-sample *t*-tests. Graphs represent mean ± SEM. Single values are represented as data points. ^****^p < 0.0001;^***^p < 0.001; ^**^p < 0.01; ^*^p < 0.05.

Next, to differentiate between Atp8a2 expression and its flippase activity, we designed a peptide inhibitor targeting Atp8a2’s interaction with CDC50A, its essential cofactor [20]. Residue-conservation mapping of the high-confidence AlphaFold2-multimer (AF2m) model of the mouse Atp8a2-CDC50A complex revealed two short, poorly conserved, surface-exposed loops in Atp8a2 (residues 953–955 and 1009–1024) that contact the exocytoplasmic domain of CDC50A (residues 205–217) (Fig. 4A–C). Because these loops exhibit limited sequence conservation and diverge markedly from Atp8a1, they provide an ideal isoform-selective target for inhibiting Atp8a2-CDC50A interaction (Supplementary Fig. 3; please see “Methods” for details). We therefore grafted CDC50A residues 205–217 onto a TAT cell-penetrating peptide, separated by a flexible GAG linker, yielding the inhibitory peptide PI8a2 (GRKKRRQRRRPQ-GAG-GIAWWTDKNVKFR). PI8a2 is predicted to bind the extracellular loops of Atp8a2, displace CDC50A, and acutely dampen flippase activity. A scrambled control sequence (scr-PI8a2; GRKKRRQRRRPQ-GAG-GIAKWTRKAVEFD) was designed in parallel.

Proximity ligation assay (PLA) analysis demonstrated that PI8a2 effectively disrupts the physical interaction between Atp8a2 and CDC50A, as indicated by a reduction in PLA puncta compared to scr-PI8a2 (Fig. 4D, E). Treatment with PI8a2 caused a marked and progressive increase in PS exposure compared to the control (Fig. 4F, G), consistent with our observations of elevated PS externalization in neurons with reduced Atp8a2 expression (Fig. 3). Non-linear regression analysis using a one-phase association model, assuming that increased PS exposure correlates with effective enzyme inhibition, showed that PI8a2 treatment generated a steep ascending sigmoidal curve with a high-rate constant (Kτ), indicative of strong Atp8a2 activity inhibition (Fig. 4H). In contrast, scr-PI8a2 gave a flatter curve with a lower Kτ, confirming no inhibition. Interaction with CDC50A, in addition to modulating Atp8a2 activity, may also influence its localization on the cell surface [20]. To address this issue, we used the membrane-impermeable protease chymotrypsin as a surface expression assay [42]. Neurons were treated with PI8a2 or scr-PI8a2 and then exposed to chymotrypsin proteolysis, and we consequently measured the amount of surface Atp8a2. Uncleaved Atp8a2, corresponding to the intracellular content in the presence of chymotrypsin, showed comparable patterns in neurons after PI8a2 or scr-PI8a2 treatment (Fig. 4I, J). Tubulin, an intracellular protein, remained uncleaved with or without exposure to chymotrypsin. This confirms that Atp8a2 remains at the plasma membrane upon disruption of its interaction with CDC50A and interfering with its activity. Overall, these findings suggest Atp8a2 flippase activity, in addition to its expression, as key in maintaining PS asymmetry in mature neurons.

### Spatial PS regulation requires Atp8a2 expression rather than activity alone

**Fig. 5:**
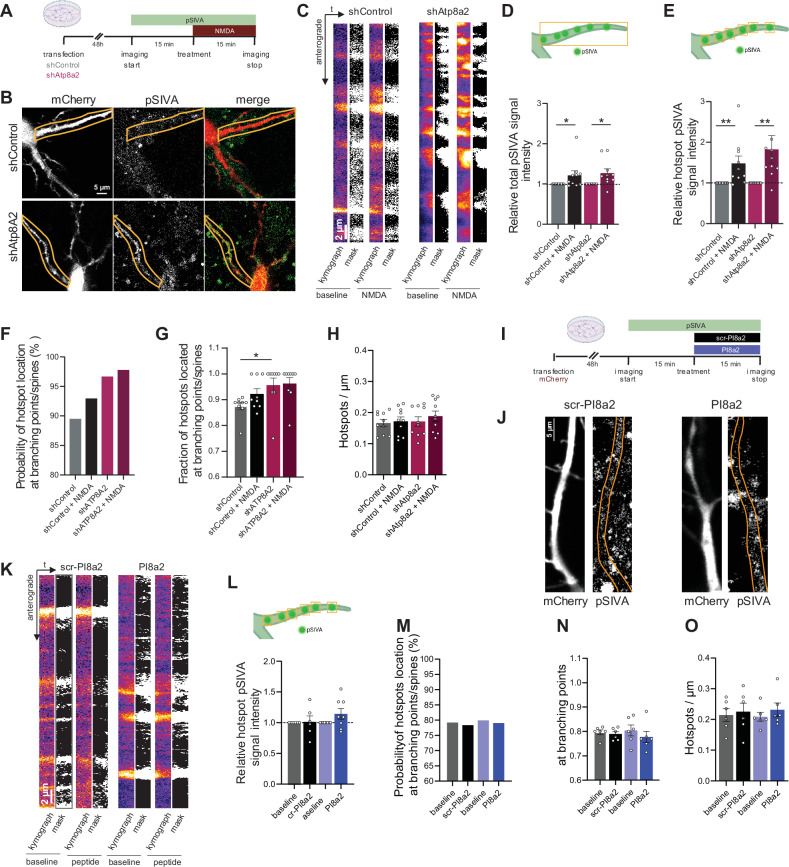
Atp8a2 expression but not its flippase activity impacts spatial organization of externalized PS. **A**–**H** Live imaging of dendritic PS exposure in shAtp8a2- and shControl-transfected hippocampal neurons ± NMDA treatment. **A** Experimental procedure. Icon created in BioRender. Mauceri, D. (2026) https://BioRender.com/qbpkcnn. **B** Representative proximal dendrites of shControl- or shAtp8a2-transfected neurons with pSIVA signals at 10 min baseline. Analyzed dendrites in yellow. Scale bar = 5 µm. **C** Representative kymographs and binary masks of pSIVA signal from (**B**) during 15 min baseline and 15 min post-NMDA. Scale bar = 2 µm. Relative pSIVA fluorescence intensity of **D** total dendrite area or (**E**) within hotspots, normalized to baseline. Icons created in BioRender. Mauceri, D. (2026) https://BioRender.com/x032fp9. **F**–**H** Analysis of pSIVA hotspots pattern. **F** Probability of hotspots at dendritic branching points/spines relative to baseline (mixed logistic regression). **G** Fraction of hotspots at dendritic branching points/spines over total hotspots. **H** Hotspot density along dendrites. *N* = 5 independent cultures, 10 neurons, 1-2 dendrites per neuron. One-sample *t*-test (shAtp8a2 / NMDA in **D**) and Wilcoxon signed-rank test (shControl / NMDA in **D**, all in **E**) for comparison to baseline; One-way ANOVA with Bonferroni’s post hoc test (**G**, **H**). **I**–**O** Live imaging of dendritic PS exposure in mCherry-transfected hippocampal neurons ± PI8a2 / scr-PI8a2 treatment. **I** Experimental procedure. Icon created in BioRender. Mauceri, D. (2026) https://BioRender.com/qbpkcnn. Representative images (**J**) and corresponding kymographs (**K**) of pSIVA live imaging in high-magnification of an mCherry-transfected proximal dendrite of a hippocampal neuron, 10 min after peptide treatment. Dendritic region within pSIVA signal is highlighted in yellow. Scale bars: images = 5 µm; kymographs = 2 µm. **L** Relative pSIVA fluorescence intensity within hotspots, normalized to baseline. Icon created in BioRender. Mauceri, D. (2026) https://BioRender.com/x032fp9. **M**–**O** Hotspots pattern analysis. **M** Probability of hotspots at branching points/spines relative to baseline (mixed logistic regression). **N** Fraction of hotspots at dendritic branching points/spines over total hotspots. **O** Hotspot density along dendrites. *N* = 3 independent cultures, 5-6 neurons, 1-2 dendrites per neuron. One-sample *t*-test (**L**) for comparison to baseline; One-way ANOVA with Bonferroni’s post hoc test (**M**, **O**). Graphs display mean ± SEM. Single values are represented as data points. ^**^p < 0.01; ^*^p < 0.05.

Next, we examined if the loss of Atp8a2 expression or activity impacts the spatial dynamics of PS exposure (Fig. 5A). Excitotoxicity increased the total PS externalization in dendrites of both Atp8a2-deficient and control (shControl) neurons compared to baseline (Fig. 5B–D) and specifically in discrete hotspots along dendrites (Fig. 5C, E). Interestingly, reduced Atp8a2 expression alone significantly increased the likelihood of PS hotspots forming at dendritic branching points and spines, mimicking the effect of NMDA treatment (Figs. 1 and 5F, G). NMDA application in Atp8a2-deficient neurons did not further alter hotspot localization, suggesting that the absence of Atp8a2 saturates the redistribution of PS exposure (Fig. 5G) and the overall density of PS hotspots along dendrites remained unchanged (Fig. 5H). This indicates that the loss of Atp8a2 expression redistributes existing PS hotspots towards structural dendritic features in a manner similar to excitotoxicity.

Next, we assessed the impact of interfering with Atp8a2 activity on PS hotspots (Fig. 5I, J). As previously observed, baseline PS exposure was clustered in hotspots localized to branching points and spines (Fig. 5J, K). Intriguingly, neither treatment with PI8a2 or scr-PI8a2 altered the localization, number, or intrinsic PS exposure levels of these hotspots (Fig. 5L–O), indicating that the spatial distribution of PS exposure is specifically regulated by Atp8a2 expression levels rather than merely its enzymatic activity.

These experiments thus reveal that while both Atp8a2 expression and flippase activity regulate PS externalization levels, the spatial reorganization of PS exposure at certain structural sites is specifically governed by Atp8a2 expression rather than its catalytic activity.

### Atp8a2 expression is essential for structural integrity in mature neurons

**Fig. 6:**
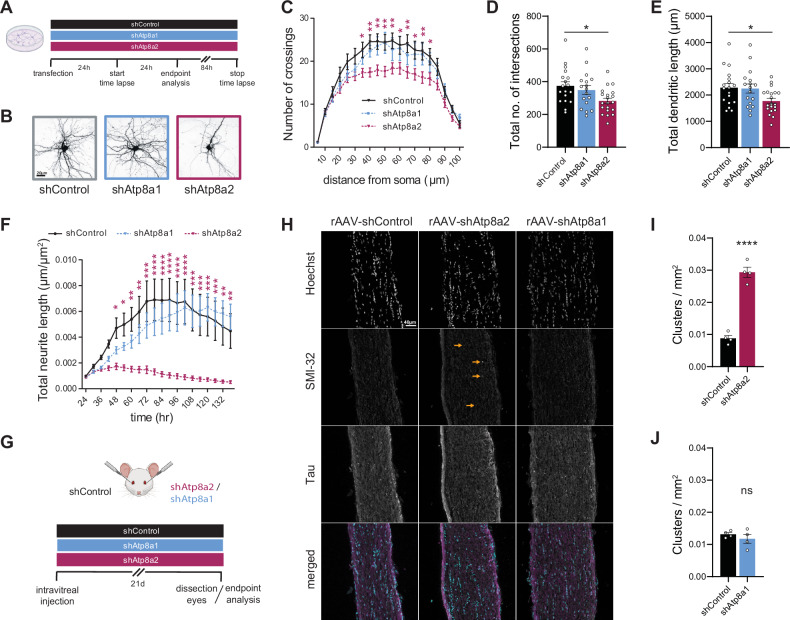
Atp8a2 expression is crucial for neuronal structural integrity in vitro and in vivo. **A** Schema of the experimental procedures. Icon created in BioRender. Mauceri, D. (2026) https://BioRender.com/qbpkcnn. **B**–**E** Morphometric analyses of hippocampal neurons co-transfected with hrGFP (vector) and shControl, shAtp8a1 or shAtp8a2. **B** Representative images of transfected neurons. hrGFP visualizes neuronal structure. Scale bar = 20 µm. Sholl analysis (**C**), total intersections (**D**), and total dendritic length (**E**) of transfected neurons. *N* = 17–20 neurons from 4 independent cultures. Two-way ANOVA with repeated measures with Dunnett’s post hoc test (**C**), or One-way ANOVA with Tukey’s post hoc test (**D**, **E**). **F** Time-lapse analyses of neurite length of hippocampal neurons transfected with shControl, shAtp8a1 or shAtp8a2. Graph shows relative total neurite length over time, beginning 24 h after transfection. *N* = 5 independent cultures. Two-way ANOVA with repeated measures with Dunnett’s post hoc test. **G**–**J** Immunohistochemical analyses of axonal disintegration in optic nerves of mice intravitreally injected with rAAV-shControl, -shAtp8a2, or -shAtp8a1. **G** Experimental setup. Icon created in BioRender. Mauceri, D. (2026) https://BioRender.com/veis2x4. **H** Representative images of optic nerves from intravitreally injected eyes and immunostained with SMI-32 (marker for dephosphorylated neurofilaments), and Tau to visualize the tissue. Nuclei labeled with Hoechst. Yellow arrows indicate SMI-32-positive clusters. Scale bar = 40 µm. Analyses of SMI-32 positive clusters in optic nerves from eyes injected with rAAV-shAtp8a2 (**I**), or rAAV-shAtp8a1 (**J**) compared to rAAV-shControl. 3-4 sections per condition, *N* = 4 mice. Two-tailed unpaired *t*-tests. Graphs represent mean ± SEM. Single values represented as data points. ^****^p < 0.0001; ^***^p < 0.001; ^**^p < 0.01; ^*^p < 0.05; ns non-significant *p* > 0.05.

During neuronal development, reduced Atp8a2 expression impairs neurites and axonal growth [25, 26], but whether Atp8a2 is also essential for maintaining the structural integrity of mature neurons remains unknown. We sought to address this question for two reasons: first, because our data link several neurodegenerative conditions to Atp8a2 loss; and second, because PS externalization — regulated by Atp8a2 — appears preferentially at structural domains such as branching points, suggesting possible vulnerability in these regions. Indeed, PS exposure in specific spatial subdomains has been linked to degenerative processes [43, 44]. shAtp8a2-transfected mature neurons showed an overall reduction in total dendritic length and complexity compared to shControl-transfected neurons, indicative of a destabilization of dendritic stability characteristic of early neurodegeneration (Fig. 6A–E) [45, 46]. No difference was observed between shControl and shAtp8a1-transfected neurons (Fig. 6A–E). Automated, continuous, non-invasive imaging confirmed these shAtp8a2-dependent specific structural deficits (Fig. 6F).

To validate the findings that Atp8a2 expression is essential to maintain the integrity of neuronal structure in vivo, we intravitreally injected adult WT mice with rAAV-shAtp8a2, rAAV-shAtp8a1 or rAAV-shControl (Fig. 6G) [30]. Three weeks post injection, optic nerves from mice with reduced Atp8a2 expression showed markedly elevated levels of dephosphorylated neurofilaments (SMI-32 clusters), indicating axonal stress and damage characteristic of neurodegenerative pathology (Fig. 6H, I) [30]. No such effect was observed with Atp8a1 knockdown (Fig. 6H, J).

As Atp8a2 loss drives PS externalization specifically at branching points (Fig. 5G) and compromises neuronal structural integrity (Fig. 6), we asked whether Atp8a2 might associate with cytoskeletal and/or structurally-relevant protein networks. Due to the current lack of validated antibodies suitable for endogenous-specific immunoprecipitation of murine Atp8a2, we systematically reanalyzed previously published interactomic datasets of Atp8a2 derived from multiple experimental systems, namely bovine retinal tissue and heterologous HEK293 overexpression models [47]. Gene Ontology (GO) enrichment analyses consistently identified cytoskeleton-associated pathways among the most significantly enriched categories, with STRING network analysis further highlighting a consistent set of cytoskeletal and synaptic scaffold proteins including spectrin (SPTBN1, SPTAN1), ankyrin-2 (ANK2), microtubule-associated proteins (MAP4, MAP1B), Band 4.1-like protein (EPB41L2), and CaMKII (CAMK2D) (Supplementary Fig. 4A–D). To corroborate this biochemically, subcellular fractionation of adult hippocampal tissue revealed substantial enrichment of Atp8a2 in synaptosomal, Triton-insoluble and postsynaptic density fractions, while Atp8a1 showed markedly weaker association with these same compartments (Supplementary Fig. 4E).

These results establish that Atp8a2 expression is essential for maintaining structural integrity in fully developed neurons, possibly via interactions with the cytoskeleton or structurally relevant proteins, extending its known roles beyond developmental contexts to the maintenance of mature neuronal architecture.

### Atp8a2 expression determines neuronal survival independently of enzymatic activity

**Fig. 7:**
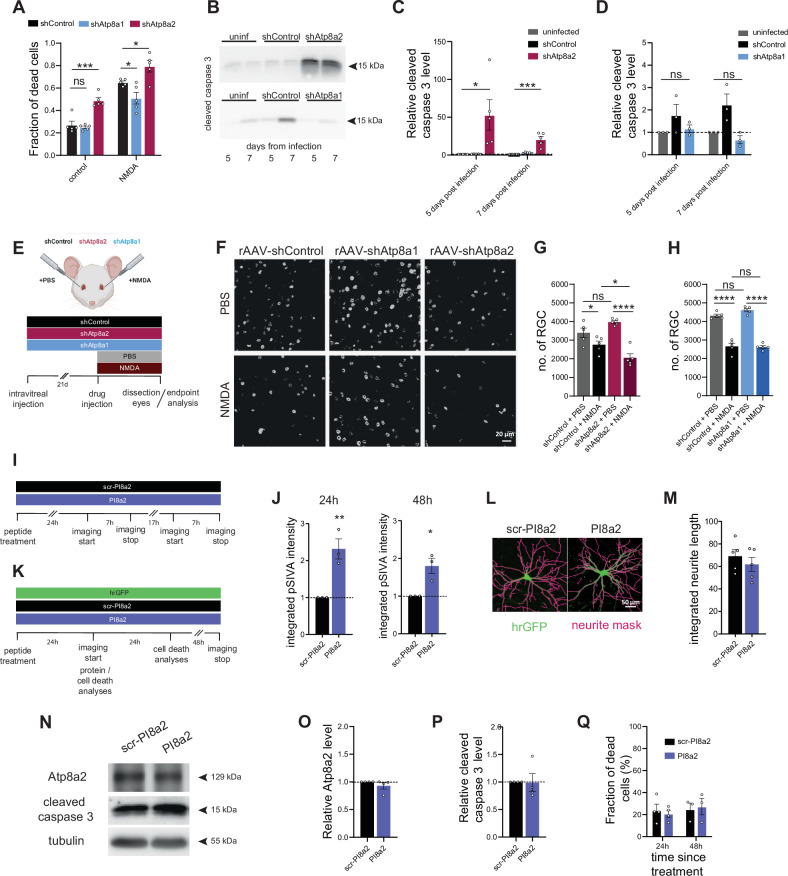
Loss of Atp8a2 increases neuronal vulnerability in vitro and in vivo. **A** Cell death analyses of shAtp8a2-, shAtp8a1-, or shControl-transfected hippocampal neurons ± NMDA treatment. Quantification of cell death as percentage of pyknotic/total neuronal nuclei. *N* = 5 independent cultures. Two-way ANOVA followed by with Dunnett´s post-hoc test. **B**–**D** Immunoblot analyses of cleaved caspase 3 in hippocampal neurons infected with shAtp8a2, shAtp8a1, or uninfected for 5–7 days. **B** Representative immunoblots of cleaved caspase 3 for shAtp8a2, shAtp8a1, and respective shControl 5 or 7 days after infection. Cleaved caspase 3 levels normalized to tubulin and uninfected control for shAtp8a2 (**C**) or shAtp8a1 (**D**) after 5 or 7 days post-infection. *N* = 3-5 independent cultures. One-way ANOVA with Dunnett’s post hoc test. **E**–**H** Cell death analyses of RGCs infected with rAAV-shAtp8a2, -shAtp8a1, or -shControl and treated with PBS (vehicle) or NMDA. **E** Experimental procedure. Icon created in BioRender. Mauceri, D. (2026) https://BioRender.com/veis2x4. **F** Representative images of whole-mounted retinas from intravitreally injected eyes, immunostained with Brn3a (RGC marker). Scale bar = 20 µm. Quantification of RGCs number for shAtp8a2 (**G**) or shAtp8a1 (**H**) infection ± treatment. N = 4–5 mice. Two-way ANOVA with Bonferroni´s post-hoc test. **I**–**Q** Long-term PI8a2 or scr-PI8a2 treatment. **I** Timeline of experiment (**J**). **J** Integrated pSIVA fluorescence intensity 24 or 48 h post-peptide treatment. Acquisition every hour for 7 h. *N* = 3 independent cultures. Two-tailed unpaired *t*-test. **K** Schema of the experiments. **L**, **M** Time-lapse imaging of neurite length for 3 days of hippocampal neurons transfected with hrGFP, with peptide treatment. **L** Representative images of hrGFP-expressing neuron after 4 days of treatment overlaid with automatically generated neurite mask used to measure neurite length. Scale bar = 50 µm. **M** Integrated total neurite length over time normalized to first timepoint, beginning 24 h after transfection. *N* = 5 independent cultures. Two-tailed unpaired *t*-test. **N**–**P** Immunoblot analyses of Atp8a2 and cleaved caspase 3 in hippocampal neurons with 24 h PI8a2 or scr-PI8a2 treatment. **N** Representative immunoblots. Atp8a2 (**O**) or cleaved caspase 3 (**P**) levels normalized to tubulin and control. *N* = 4 independent cultures. One-sample *t*-test. **Q** Cell death analyses of hippocampal neurons with PI8a2 or scr-PI8a2 treatment for 24 h or 48 h. *N* = 3–4 independent cultures. Two-way ANOVA with Bonferroni´s post-hoc test. Graphs display mean ± SEM. Single values are represented as data points. ^****^p < 0.0001; ^***^p < 0.001; ^*^p < 0.05; ns non-significant *p* > 0.05.

Reduced Atp8a2 expression in neurons increases PS exposure on the outer plasma membrane in discrete domains and compromises structural stability (Figs. 5 and 6); both early events of neuronal degeneration [7, 46]. Moreover, we found that neurons with reduced Atp8a2 exhibited a significantly higher mortality rate, which worsened over time (Fig. 7A). This correlated with dramatically increased levels of cleaved caspase 3 (Fig. 7B, C), a widely recognized marker of cell death [48, 49]. In contrast, Atp8a1 knockdown had no such effect (Fig. 7A, B, D). When challenged with NMDA toxicity [46], Atp8a2-deficient neurons showed exacerbated cell death, while Atp8a1 knockdown conferred no additional vulnerability (Fig. 7A). We confirmed these findings in vivo by inducing NMDA-triggered excitotoxicity in mice with rAAV-mediated knockdown of Atp8a2 or Atp8a1 in adult RGCs (Fig. 7E). Consistent with our in vitro results, reduced Atp8a2 significantly increased RGC vulnerability to the toxic insult compared to controls (shControl and shAtp8a1; Fig. 7F–H). These findings underscore the critical role of Atp8a2 expression in neuronal vulnerability.

Our results showed that reduced Atp8a2 expression altered spatial PS exposure (Fig. 5A–E) and changed its pattern (Fig. 5F–H) with consequences on neuronal structural integrity and viability (Figs. 6 and 7A–G). Inhibition of Atp8a2 activity, however, led to PS exposure without affecting its spatial distribution (Fig. 5I–O). To test whether inhibition of Atp8a2 activity would affect neuronal structural integrity and viability, we treated neurons with PI8a2 for an extended period (Fig. 7I). Prolonged treatment with PI8a2 caused sustained PS externalization at both 24 and 48 h (Fig. 7J). However, despite this robust and persistent PS exposure, PI8a2-treated neurons maintained normal dendritic architecture compared to scr-PI8a2 controls (Fig. 7K–M). This preservation of structural integrity stands in strong contrast to the profound structural degeneration observed in Atp8a2-deficient neurons (Fig. 6), despite comparable PS externalization. Further, prolonged inhibition of Atp8a2 activity with PI8a2, despite still being functional and causing PS externalization (Fig. 7J), did not affect Atp8a2 protein levels (Fig. 7N). This confirms that PI8a2 affects Atp8a2 activity without influencing its expression. Most importantly, neuronal viability remained unchanged even after 48 h of sustained PS exposure (Fig. 7P, Q). Taken together, these findings indicate that Atp8a2 expression, rather than its enzymatic activity alone, is essential for neuronal integrity and survival.

### Increased levels of Atp8a2 are neuroprotective

**Fig. 8:**
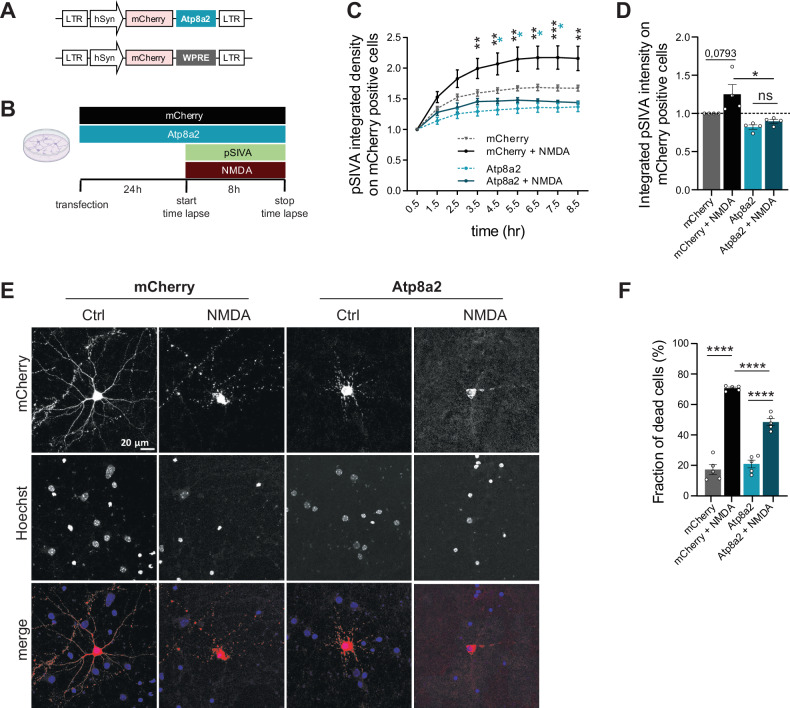
Increased Atp8a2 expression mitigates PS exposure and neuronal death. **A**–**D** Time-lapse analyses of pSIVA signal in mCherry- or Atp8a2-transfected hippocampal neurons ± NMDA treatment. **A**, **B** Constructs and experimental procedure. Icon created in BioRender. Mauceri, D. (2026) https://BioRender.com/qbpkcnn. Relative pSIVA integrated density on mCherry-positive neurons over time (**C**) and integrated (**D**). pSIVA signal is normalized to initial timepoint and untreated control. *N* = 4 independent cultures. Two-way ANOVA with repeated measures with Dunnett’s post hoc test for over time analyses (**C**), or One-way ANOVA with Bonferroni’s post hoc test for integrated analyses (**D**). **E**, **F** Analysis of cell death of mCherry- or Atp8a2-expressing hippocampal neurons. **E** Representative images of mCherry- or Atp8a2-transfected neurons ± NMDA treatment. Nuclei were visualized with Hoechst. Scale bar = 20 µm. **F** Quantification of cell death as percentage of pyknotic/total neuronal nuclei. *N* = 5 independent cultures. One-way ANOVA followed by with Bonferroni’s post hoc test. Graphs display mean ± SEM. Single values are represented as data points. ^****^p < 0.0001; ^***^p < 0.001; ^**^p < 0.01; ^*^p < 0.05; ns non-significant *p* > 0.05; (minimal significant p values are displayed).

Finally, given the key role of Atp8a2 expression in determining neuronal integrity and survival, we investigated whether enhanced Atp8a2 expression could confer neuroprotection. We overexpressed Atp8a2 in mature hippocampal neurons and assessed PS asymmetry under basal and excitotoxic conditions (Fig. 8A). Atp8a2 overexpression significantly reduced baseline PS externalization compared to control neurons, demonstrating enhanced maintenance of membrane asymmetry under physiological conditions (Fig. 8C, D). When challenged with NMDA, control neurons exhibited the expected robust increase in PS exposure, while Atp8a2-overexpressing neurons maintained near-baseline levels of PS asymmetry throughout the excitotoxic insult (Fig. 8C, D). Most importantly, Atp8a2 overexpression rendered neurons significantly more resistant to excitotoxic cell death (Fig. 8E, F).

In sum, our findings reveal that Atp8a2 expression levels — distinct from its PS modulation activity — critically determine neuronal vulnerability to structural damage and degeneration in mature neurons, underscoring its pivotal role in maintaining neuronal homeostasis and identifying a novel therapeutic target for neurodegenerative disorders.

## Discussion

Our findings establish a fundamental principle: PS externalization is necessary but not sufficient to drive neurodegeneration. While both loss of Atp8a2 expression and pharmacological inhibition of its flippase activity trigger PS exposure, only the former drives spatial redistribution of PS to vulnerable structural sites, compromises neuronal architecture, and ultimately causes neuronal death. Importantly, Atp8a2 loss emerges as a convergent vulnerability mechanism across multiple neurodegenerative conditions, placing it at the center of a previously unrecognized mechanism of neuronal vulnerability with direct therapeutic implications.

### Glutamate toxicity drives PS externalization

PS exposure has been traditionally viewed as an unescapable apoptotic signal that attracts engulfing cells. However, mounting evidence now indicates that non-apoptotic signals can also trigger PS exposure in both neuronal and non-neuronal cells [3, 50–53]. Our findings align with these observations and show that glutamate and eNMDAR toxicity—non-apoptotic signals—significantly alter PS dynamics as they promote PS externalization and Atp8a2 downregulation. Of note, in glucose-oxygen-deprivation models, which also lead to toxic activation of eNMDAR via glutamate spillover from the synaptic cleft [11], PS exposure has been reported [54, 55]. Notably, while toxic eNMDAR activation causes calcium influx leading to Atp8a2 loss and PS exposure, Bic treatment — which also elevates intracellular calcium — does not. This suggests that these responses are specifically linked to toxic signaling rather than general calcium elevation, possibly due to differences in calcium concentration [52] or entry source [22]. Further, we reveal a novel, spatially organized pattern of PS externalization in mature neurons, with hotspots preferentially localized to dendritic branching points and spines. Loss of Atp8a2 expression — whether via toxicity or targeted RNAi knockdown — enhances this pattern, potentially destabilizing crucial structural elements and suggesting these domains are critical, vulnerable sites for membrane-cytoskeleton interactions.

### Atp8a2-dependent organization of PS hotspots at vulnerable neuronal sites

The localization of PS externalization to specific hotspots represents an intriguing feature of neuronal PS dynamics with relevance to understanding disease progression. While previous studies documented PS externalization in stressed neurons [8], our observed pattern suggests a regulated process rather than random membrane disruption. These sites may represent membrane-ER contact points where PS is delivered from its synthesis location. Alternatively, the high membrane curvature and specialized lipid composition at these locations could create microdomains favorable for PS translocation. A third possibility is that elevated calcium flux in these areas, due to concentrated receptors and channels, may influence local lipid dynamics through calcium-dependent enzymes.

The identification of discrete PS externalization hotspots also has implications for our understanding of neurodegenerative processes. These hotspots might serve as initial sites of vulnerability where structural deterioration begins, particularly given their localization at critical dendritic junctions and potential ER contact sites. This spatial organization could explain the often-observed pattern of discrete, localized degeneration in early stages of neuronal death, before widespread cellular dysfunction occurs. Previous studies have also reported PS externalization in specific compartments during degeneration [43, 44, 56], but the mechanisms governing this spatial distribution remained unknown. Our results now demonstrate that reduced Atp8a2 expression levels modulate the distribution of these PS hotspots in dendrites, raising intriguing questions about potential structural roles of Atp8a2 beyond its canonical flippase function.

### Atp8a2 supports structural integrity beyond development

We observed that Atp8a2 loss significantly impairs neuronal integrity in mature neurons, revealing a structural maintenance function distinct from its known developmental roles. Specifically, Atp8a2 depletion reduced dendritic length and complexity while also inducing quantifiable axonal damage in optic nerves. This comprehensive structural impactaffecting both dendrites and axons of different neuronal types — extends beyond the primarily axonal focus of prior literature [25, 26, 57]. Critically, these structural alterations occurred in fully developed neurons rather than during neurogenesis or neuritogenesis, establishing Atp8a2’s ongoing importance throughout neuronal lifespan. Previous investigations linking Atp8a2 to neuronal integrity have predominantly focused on developmental contexts, utilizing either conventional KO mice — affecting Atp8a2 expression from embryonic stages — or manipulations in immature cultured neurons or non-neuronal PC12 cells [16, 25, 26]. Our approach, which selectively depletes Atp8a2 in mature neurons while preserving its expression during development, reveals its essential role in ensuring that established neuronal architecture endures throughout the lifespan. When this maintenance function is compromised, structural destabilization likely contributes to neurological dysfunction in various conditions [10, 45, 58]. While Atp8a2 deficiency and mutations have been linked to several developmental dysfunctions in cognition, motor activities, and sensory processing in both mouse models and patients [16, 23, 26, 36–39], it remains an open question whether loss of Atp8a2 specifically in mature neurons would affect these systems similarly through impaired structural stability and neuronal viability. The molecular basis of this structural maintenance function remains an important open question. Reanalysis of published Atp8a2 interactomic datasets from multiple experimental systems consistently identifies cytoskeleton-associated pathways and specific synaptic scaffold proteins, including spectrin, ankyrin-2, microtubule-associated proteins, and CaMKII. Biochemical fractionation further reveals preferential enrichment of Atp8a2, but not Atp8a1, in synaptic regions. These convergent observations support a working hypothesis in which Atp8a2 maintains neuronal structural integrity through organizational roles within cytoskeletal and synaptic protein networks, and the definitive characterization of the Atp8a2 interactome represents an important avenue for future investigation.

### Functional divergence between Atp8a2 and Atp8a1 in mature neurons

Surprisingly, our findings distinguish Atp8a2 from its paralogue Atp8a1 in response to toxic insults, revealing functional specificity with important therapeutic implications. While Atp8a2 expression significantly declined following toxicity and in models of neurodegeneration, Atp8a1 remained stable. This distinction proved consequential as Atp8a2 depletion drove PS externalization, whereas Atp8a1 reduction had no such effect. Such findings contrast with previous reports of increased PS exposure in neurons derived from Atp8a1 KO mice [17], likely reflecting fundamental differences between our targeted approach in mature neurons versus conventional KO models with their inherent developmental alterations and compensatory adaptations. The functional separation between Atp8a1 and Atp8a2 extended to structural integrity, where Atp8a2 loss triggered both PS externalization and neuronal disintegration, while Atp8a1 deficiency left neurons structurally intact. Perhaps most compelling were our survival assays, which revealed that Atp8a2 deficiency compromised neuronal viability, while Atp8a1 reduction conferred subtle opposite protective effects. These results challenge the presumed functional redundancy of these flippases in neurons and highlight Atp8a2’s unique, essential role in maintaining mature neuronal integrity. From a therapeutic perspective, this specificity suggests that Atp8a2-targeted interventions are unlikely to be compensated by Atp8a1 upregulation, making Atp8a2 a more reliable therapeutic target.

### Expression versus enzymatic activity: implications for therapeutic strategy

Our findings reveal an unexpected dichotomy between Atp8a2’s flippase activity and expression levels in neuronal integrity and viability. While both reduced expression and inhibition increase PS exposure, only diminished Atp8a2 expression increases PS at vulnerable hotspots, compromises structural stability and leads to neuronal death. Atp8a2 may thus function as a scaffold protein, independent of its flippase activity, maintaining local membrane organization, lipid domain structure, crucial protein-protein interactions and/or signaling complexes affecting neuronal survival. Our observations that caspase 3 activation occurs only in neurons with reduced expression of Atp8a2 support these interpretations. Notably, different mutants of Atp8a2 not only affect flippase activity but often also result in significantly lower protein production with disrupted and unstable structure compared to non-mutated Atp8a2 [59]. Consistent with this scaffold hypothesis, reanalysis of published interactomic datasets and biochemical fractionation of hippocampal tissue identify cytoskeletal and synaptic scaffold proteins as the preferential molecular environment of Atp8a2.

The molecular response of Atp8a2 loss appears conserved across glutamate-related pathologies occurring in both acute toxic conditions and chronic neurodegeneration. These findings align with previous studies showing dysregulated glutamate signaling and eNMDAR activation as common pathological mechanisms in neurological disorders [12, 21, 22, 46]. Given that glutamate toxicity contributes to stroke, traumatic brain injury, epilepsy, Alzheimer’s disease, Parkinson’s disease, and amyotrophic lateral sclerosis, Atp8a2-based therapeutics could have broad clinical applicability. The exact mechanism of glutamate toxicity-induced Atp8a2 loss remains an important area for future investigation, with possibilities including transcriptional repression, mRNA destabilization, impaired translation, enhanced protein degradation, altered post-translational modifications, or disrupted protein trafficking. Understanding these mechanisms could reveal additional therapeutic intervention points.

Increasing Atp8a2 expression not only restores PS asymmetry but also confers significant neuroprotection against glutamate toxicity. Preserving Atp8a2 levels in the medial prefrontal cortex protects mouse pups from neurodevelopmental disorders triggered by maternal immune activation — a condition that typically reduces Atp8a2 expression in that brain region [60]. From a therapeutic perspective, maintaining Atp8a2 expression, not merely its enzymatic activity, may thus be neuroprotective. The discovery that Atp8a2 expression, rather than activity alone, is crucial for neuroprotection suggests that traditional enzyme replacement or small molecule enzyme activators may be insufficient to prevent or halt neurodegeneration. Instead, strategies that enhance or preserve Atp8a2 expression represent a more promising therapeutic direction.

In conclusion, our findings establish Atp8a2 as a novel and convergent determinant of neuronal vulnerability across neurodegenerative conditions, bridging fundamental membrane biology with disease pathology. The spatial organization of PS externalization at structurally vulnerable dendritic sites, and its specific dependence on Atp8a2 expression rather than catalytic activity, raise important questions about the full spectrum of Atp8a2 functions in mature neurons. Understanding these mechanisms represents an exciting frontier that may reveal new intervention points for neuroprotective strategies targeting Atp8a2 expression across diverse neurodegenerative diseases.

## Methods

### Hippocampal cultures, transfections and treatments

Hippocampal neurons were isolated from P0 C57Bl/6N mice and cultured as previously described [15]. DNA transfection was performed after a culturing period of 8 days in vitro (DIV) using Lipofectamine 2000 (Invitrogen) and following previously published protocols [15, 61]. Experiments were conducted on neurons at 9–10 DIV. Unless otherwise specified, cells were treated on DIV 9–10 as following: 50 μM Bicuculline (Tocris Bioscience), 20 μM NMDA (Biotrend) for 10 min or up to 6 h, 10 µM GW4869 (Sigma), 10 µM Y-27632 (Tocris Bioscience), 50 μM TBOA (Tocris Bioscience), 20 μM glutamate (Tocris Bioscience),10 μM PI8a2/scr-PI8a2 (Covalab; Synpeptide). Control cells were treated with the vehicle only.

### pSIVA imaging

Primary neurons were either transfected with the specified construct on DIV 8 and then incubated with pSIVA (1:500; Bio-Techne) and a drug/vehicle on DIV 9 or 10, or they were only treated with a peptide on DIV 10 and incubated with pSIVA (1:500; Bio-Techne). For the long-term peptide experiments, pSIVA was added after 24 or 48 h of peptide treatment. Time-lapse imaging was conducted using the Incucyte® S3 Live-Cell Analysis System (Sartorius) with image acquisition every hour for 4–8 h in both green (excitation: 441–481 nm, emission: 502–544, acquisition time: 300 ms) and red (excitation: 567–607 nm, emission: 622–704, acquisition time: 400 ms) or only green channels using a 10× objective. Sixteen images (875 × 645 μm) were obtained per well (1.9 cm², ~0.18 × 10^6^ cells). The pSIVA signal was analyzed globally or selectively in transfected cells using Incucyte® Basic Analyzer Software (Sartorius). For green channel (pSIVA) analysis, settings were Top-Hat filter (radius: 11 μm, threshold: 1–1.5 GCU); for red channel (transfection marker) Top-Hat filter (radius: 20 μm, threshold: 2 RCU, area min: 120 μm²). pSIVA fluorescence was normalized either per well to the initial timepoint or, for transfected cells, based on the overlap of pSIVA signal with transfected regions normalized to the initial red area per well. For quantifying pSIVA signal in cells expressing the Atp8a2-mCherry construct, images of mCherry-positive cells at the initial time point were processed in Fiji/ImageJ to generate a mask of the transfected area using the RenyiEntropy threshold (settings: 66-65535, raw). This mask was applied to background-subtracted green-channel images for all time points (background subtraction: rolling ball radius 50, stack mode). Integrated pSIVA density within the masked area was measured over 8 h and normalized to the initial integrated pSIVA density per well.

In an alternative (Supplementary Fig. 1), to confirm pSIVA dynamics in response to treatments, hippocampal neurons were cultured on 4-well Nunc® Lab-Tek Chamber Slide Systems with borosilicate glass bottoms. Before imaging, culture medium was replaced with a phenol red-free medium at DIV 10. A master mix containing pSIVA (1:500, Abcam), Propidium Iodide (1:1000, Abcam) and Hoechst 33342 (1:5000, Molecular Probes) was prepared and added to the cells. Time-lapse imaging was performed using a Nikon TI-high content screening (HCS) microscope (Nikon Imaging Center) equipped with a temperature- and CO_2_-controlled chamber. Drugs or vehicle were applied, and images were acquired as a z-series at 4 μm depth intervals. Using a 20x Apo VC objective, up to 28 randomly selected fields per well were imaged every 30 min. Z-stacks were processed into maximum intensity projections (MaxIPs), separated into individual channels, and the pSIVA fluorescence intensity of individual multipoints was quantified using Fiji. Background-subtracted pSIVA signal was normalized to control values and cell number per view.

### Mass spectrometry

To extract lipids for liquid chromatography-mass spectrometry LC-MS analysis, hippocampal cells were cultured in 60-mm dishes for each experimental condition, working on ice and using pre-chilled reagents. On DIV 10, cells were treated for 2 h with NMDA and harvested in PBS, followed by centrifugation at 2000 × *g* for 5 min at 4 °C. The pellets were then snap-frozen in liquid nitrogen and stored at −80 °C. Using MS-grade reagents under a cold environment, cells were lysed in methanol with internal standards (SPLASH® Lipidomix®; Avanti) for protein precipitation, then mixed with chloroform and sonicated. After adding H₂O for phase separation, samples were centrifuged, and the top aqueous layer was carefully removed. The chloroform phase was collected, dried in a speedvac (30 °C, 20 min), and stored at −80 °C for shipping on dry ice. LC-MS to quantify neuronal lipid quantity was performed by the EMBL Metabolomics Core Facility, Heidelberg, Germany.

### Analysis of pSIVA live imaging in dendrites

Time-lapse imaging of pSIVA fluorescence on transfected neurons was performed on DIV 10 at 1-min intervals using a Leica Sp8 confocal laser scanning microscope with a 63× water immersion objective and 4× optical zoom at a resolution of 1024 × 1024 pixels (0.043 µm/pixel). Imaging was performed in HEPES-buffered saline solution (HBS) containing: 140 mM NaCl, 2.5 mM KCl, 1.0 mM MgCl_2_, 2.0 mM CaCl_2_, 10 mM HEPES, 1.0 mM glycine, 35.6 mM D-glucose, and 0.5 mM Na-pyruvate, and additional pSIVA (1:500; Bio-techne). Only proximal dendrites were selected for analysis. Following a 15-min baseline recording, neuronal cultures were treated with the specified drug or peptide, and imaging was continued for an additional 15 min. Acquired images were processed with standard background subtraction (rolling ball radius 50, stack mode) to reduce noise. Dendrites were manually traced, and kymographs were generated using the KymoResliceWide plugin in ImageJ/Fiji [62]. The accumulation of pSIVA-positive signal (hotspots) was quantified by counting the number of accumulations per dendrite, including those within a 2-μm proximity to branching points or spines. Hotspot probability and distribution were assessed using a mixed logistic regression model, with the number of hotspots per cell as the unit of analysis, incorporating factors such as genetic modification and pharmacological treatment. Data analysis was performed with R 3.6.3 (R Foundation for Statistical Computing). The R script used for mixed logistic regression analysis is available upon request. The mean grey value of hotspots was quantified within defined regions of interest (ROIs) following the application of Gaussian blur (*σ* = 0.7) and Renyi Entropy thresholding. Particles sized ≥40 pixels with circularity between 0 and 3 were recognized. Grey values were normalized to untreated baseline conditions for comparison.

### QRT-PCR

Total RNA was extracted using the RNeasy Mini Kit (Qiagen) including an optional DNase I treatment at room temperature for 15 min according to manufacturer’s instructions (Qiagen). Extracted RNA was reverse transcribed into first-strand cDNA using the High-Capacity cDNA Reverse Transcription kit (Applied Biosystems). Quantitative reverse transcriptase PCR (qRT-PCR) was done on a StepOne plus Real Time PCR system using TaqMan Gene Expression Assays for the indicated genes (Applied Biosystems). The following TaqMan Gene Expression Assays were used in this study: Atp8a2 (Mm00443740_m1), Atp8a1 (Mm00437712_m1), Plscr4 (Mm00453379_m1), Atp11a (Mm00443760_m1), Atp11c (Mm01297974_m1), Abca1 (Mm00442646_m1), Tmem16f (Mm00614693_m1), Atp8b2 (Mm01220121_m1), Plscr1 (Mm01228223_g1), Plscr3 (Mm00840221_m1), Xkr4 (Mm02343615_m1), Xkr8 (Mm01187363_m1), Cdc50a (Mm00505138_m1), cFos (Mm00487425_m1), and Gusb (Mm00446953_m1) or Gapdh (Mm99999915_g1) as endogenous control genes for normalization.

### Western blotting

Primary hippocampal cultures were lysed directly in Laemmli buffer (30% glycerol, 4% SDS, 160 mM Tris-HCl pH 6.8, 0.02% bromophenol blue, 10 mM DTT) and heated at 95 °C for 10 min. Western blotting was performed according to standard procedures. The primary antibodies were used in 5% milk and included rabbit anti-Atp8a2 (1:6000, Santa Cruz Biotechnology), mouse anti-tubulin (1:400,000, Merck Millipore), and rabbit anti-Atp8a1 (1:6000), kindly provided by Dr. Xiao-Song Xie, 5% BSA including rabbit anti-Atp8a2 (1:500, Origene), mouse anti-caspase 3 (1:1000, Santa Cruz Biotechnology), and rabbit anti-cleaved caspase 3 (1:1000, Cell Signaling), or I-Block™ (Thermo Fisher) including rabbit anti-Atp8a1 (1:1000, Proteintech), mouse anti-PSD-95 (1:1000, Abcam), rabbit anti- Synaptophysin (1:100,000, Abcam), and rabbit anti-vGlut1 (1:10,000, Abcam). The secondary antibodies were either goat anti-mouse IgG (H+L) peroxidase and goat anti-rabbit IgG (H+L) peroxidase (1:5000; AffiniPure Jackson Immuno Research) applied in 5% milk or goat anti-mouse IgG (H+L) peroxidase (1:10,000) and goat anti-rabbit IgG (H+L) peroxidase (1:100,000) (Bio-Rad) applied in I-Block™ (Thermo Fisher). Membranes were developed using enhanced chemiluminescence (ECL) solution (BioRad) and imaged with a ChemiDoc system (BioRad). Relative protein levels were normalized to tubulin and control samples for quantification. Images of the uncropped Western blot membranes used in the figures can be found in supplementary material.

### Intravitreal injections

For intravitreal injections, mice were anesthetized via intraperitoneal injection of 0.3 mg/kg body weight (BW) medetomidine (Alvetra und Werfft), 3 mg/kg BW midazolam (Hameln Pharmaceuticals), and 0.03 mg/kg BW fentanyl (Janssen Pharmaceutica) to induce deep anesthesia. 2 µL of rAAVs were injected intravitreally with a 32-gauge needle (NanoFil syringe, 10 mL; World Precision Instruments) under a stereomicroscope. Following the injection, eye ointment (Bepanthen; Roche) was applied to protect the cornea. Anesthesia was antagonized by 450 mg/kg BW atipamezole (Prodivet Pharmaceuticals), 0.3 mg/kg BW flumazenil (Fresenius Kabi), and 0.7 mg/kg BW narcanti/naloxone (Inresa Arzneimittel). For experiments involving rAAV-shAtp8a2, rAAV-shAtp8a1, and rAAV-shControl, a 21-day incubation period post-injection was sufficient to achieve effective knockdown of target proteins, as previously reported [30]. To induce excitotoxicity in the retina, 10 nmol NMDA was injected intravitreally, while the contralateral eye received an equal volume of 1x PBS as a control [29, 30].

### Kainate seizure mouse model

Mice received an intraperitoneal injection of 20 mg/kg BW kainate from a stock solution of 5 mg/ml. After the indicated time point, mice were euthanized; hippocampi quickly dissected and snap frozen and stored at −80 °C until RNA extraction.

### Alzheimer’s disease mouse model

The transgenic line B6; C3-Tg (APPswe, PSEN1dE9)85Dbo (APP/PS1) was bred in-house, and male/female transgenic mice at the ages of 4, 6 and 9 months were used for assessing Atp8a2 and Atp8a1 expression. APP/PS1 are double-transgenic mice expressing a chimeric mouse/human APP (Mo/HuAPP695swe) and a mutant human presenilin 1 (PS1-dE9) [33]. At the indicated ages, mice were euthanized, hippocampi quickly dissected and snap frozen and stored at −80 °C. Total RNA was extracted in TRIZOL (Thermo Fisher) following the manufacturer’s instructions.

### Expression constructs

The following constructs were used in this study: pAAV-CMV>*hrGFP*, pAAV-U6>*shControl*, pAAV-U6>*shAtp8a1*, pAAV-U6>*shAtp8a2*, pRRL-hsyn>*mCherry-Atp8a2*, and pRRL-hsyn>*mCherry*. pAAV-CMV>*hrGFP* and pAAV-U6>*shControl* have been characterized previously [61]. Short hairpin RNAs (shRNAs) targeting Atp8a2 or Atp8a1 were cloned into an expression vector containing the U6 promoter driving shRNA expression and a CaMKII promoter driving mCherry expression. For *Atp8a2*, two distinct shRNA sequences were used: shAtp8a2^632^ 5′-ATGGCATGTGGCACACTAT-3′, targeting Atp8a2 as described in [25], and shAtp8a2^1789^ 5′-GACATTCGGGATCCTCAAT-3′ previously validated in [40]. The shRNA targeting Atp8a1 used the sequence 5′-ACCTCAAAGTACAAAGAAATC-3′, as described in [63]. The cDNA sequence encoding bovine Atp8a2 was subcloned into a pRRL vector under the control of a′ human synapsin (hsyn) promoter and N-terminally tagged with mCherry. A control pRRL vector expressing mCherry under the hsyn promoter was used as control.

### Recombinant adeno-associated viruses

rAAVs 1/2 were produced by co-transfection of HEK293 cell by standard calcium phosphate precipitation. HEK293 cells were grown in high-glucose-containing (4.5 g/liter) Dulbecco’s Modified Eagle Medium (DMEM; Life Technologies) supplemented with 10% fetal bovine serum, 100 units/ml penicillin and 100 µg/ml streptomycin (Sigma) and tested for mycoplasma contamination. Packaging of rAAVs was carried out with helper plasmids pF∆6, pRV1 and pH21 together with either pAAV-*shAtp8a2, pAAV-shAtp8a1*, or pAAV-*shControl*. Cells were collected by low-speed centrifugation, resuspended in 100 mM NaCl-10 mM Tris-HCl (pH 8.5) and lysed by incubation with 0.5% sodium deoxycholate followed by freeze-thaw cycles. rAAVs were purified using heparin affinity columns (HiTrap Heparin HP; GE Healthcare). rAAVs stocks were concentrated using Amicon Ultra-4 centrifugal filter devices (Millipore). The integrity and purity of viral particles were verified by SDS-PAGE. Infection of neuronal cultures with rAAVs was performed on DIV 3. Infection rates were determined by analyzing mCherry fluorescence and ranged from 80% to 95% of the neuronal population.

### Analysis of NBD-PS in the inner leaflet

NBD-PS (16:0-12:0 NBD-PS 1-palmitoyl-2-{12-[(7-nitro-2-1,3-benzoxadiazol-4-yl)amino]dodecanoyl}-sn-glycero-3-phosphoserine (ammonium salt; SKU:810193 P) was dissolved in methanol to get a stock solution of 10 mg/ml and aliquoted and frozen at −20 °C. Hippocampal neurons were infected at DIV3 with rAAV-shAtp8a2, rAAV-shAtp8a1, rAAV-shControl or left uninfected. Experiments were performed at DIV9-10. Prior to experiments, the infection rates - via mCherry fluorescence - were monitored. Neurons were incubated with 10 µg/ml of NBD-PS and then fluorescence was measured every 10 min for a total of 30 min in a CLARIOstar Plus microplate reader (BMG Labtec; excitation 461, emission 534) at 37 °C and 5% CO_2_. To quench fluorescence of the outer leaflet of the plasma membrane, cells were washed multiple times with 25 mM sodium dithionite on ice and afterwards returned to culturing medium and to the plate reader for measuring NBD-PS fluorescence derived from the inner leaflet of the plasma membrane.

### Computational modeling and peptide design

We retrieved the FASTA sequences for *Mus musculus* Atp8a2 and CDC50A proteins from the UniProt database (accession numbers P98200 and Q8VEK0, respectively). The three-dimensional (3D) Atp8a2-CDC50A complex was modeled using AlphaFold2-multimer (AF2m) prediction in combination MMseqs2 software, launched via Google Colab (ColabFold v1.5.2-patch) [64]. We employed the PDB100 database for the template model searching, which is curated by searching representative structures using Foldseek (https://github.com/steineggerlab/foldseek) [65] against the AlphaFold Protein Structure Database. All PDB templates identified in this process are reported in Supplementary Fig. 3A. We generated three unrelaxed models, and the top-ranked model achieved a mean predicted local distance difference test (pLDDT) score of 85.6, a predicted template modeling (pTM) score of 0.85, and an interface predicted template modeling (ipTM) score of 0.914 (Supplementary Fig. 3B), all indicative of high model reliability. Specifically, pLDDT scores above 70 reflect reliable backbone predictions, while pTM and ipTM scores above 0.5 and 0.8, respectively, suggest meaningful structural predictions and high-confidence interface modeling. These metrics confirmed that the contact interfaces between CDC50A and Atp8a2 were predicted with high confidence. To evaluate the reliability of the AF2m-predicted complex, we superimposed it onto the closely related *Homo sapiens* Atp8a1-CDC50A complex (PDB ID: 6K7J) [66] using ChimeraX [67]. The Root Mean Square Deviation (RMSD) between the two structures was 1.27 Å, confirming a high degree of structural similarity and supporting the model’s suitability for subsequent analyses.

To refine the complex structure and mimic the native environment of the proteins, we generated a membrane system embedding the Atp8a2-CDC50A complex in a bilayer membrane. The complex was integrated into a mammalian plasma membrane using the CHARMM-GUI web server [68, 69]. Subsequently, the system was placed in a water box and neutralized with 0.15 M NaCl ions, also using CHARMM-GUI. The system was parameterized using the CHARMM36m force field [70] and prepared for energy minimization employing the GROMACS Molecular Dynamics simulation software [71]. The minimization protocol comprised a combination of Steepest Descent and Conjugate algorithms to ensure convergence [72], with parameters optimized for step size and energy tolerance. This approach effectively reduced potential structural clashes, facilitated solvent accommodation around the complex, and refined atomic interactions, driving the system to a lower-energy, more realistic conformation.

Since CDC50A can also interact with Atp8a1, we next evaluated residue-level conservation in Atp8a2 to guide the design of a CDC50A-derived peptide that preferentially targets Atp8a2 over Atp8a1, despite their high sequence similarity (80.4%). We first collected homologous sequences from diverse eukaryotic flippases (Supplementary Fig. 3C). We then performed a multiple sequence alignment (MSA) with the MUSCLE algorithm (default settings; Supplementary Fig. 3D) [73–75]. Including this broad sequence set provided the phylogenetic depth needed for robust conservation estimates and detection of isoform-specific signatures. To pinpoint the exact surface residues that diverge between the two isoforms, and are therefore most likely to confer selective binding, we projected conservation scores onto the molecular surface of Atp8a2 (Supplementary Fig. 3E). Conservation scores were calculated via the entropy-based AL2CO algorithm, applying independent counts and a gap fraction of 0.5 [76]. This integration allowed us to visually inspect less conserved regions of Atp8a2 and identify areas that diverge from its paralog Atp8a1. These scores, derived from the previously generated MSA, were integrated into the protein structure using ChimeraX [67]. This analysis identified two poorly conserved extracellular regions of Atp8a2: residues 953–955 and 1009–1024 (UniProt ID: P98200).

To further confirm the conservation results and emphasize less conserved regions critical for subsequent peptide design, we employed ConservFold. This tool, executed through a precompiled Jupyter notebook on Google Colab [64, 77], provides an automated conservation analysis workflow. The limited conservation of Atp8a2 residues 953–955 and 1009–1024 (Supplementary Fig. 3F, G), and especially their divergence from the corresponding residues in Atp8a1 (Supplementary Fig. 3D), highlight their potential as Atp8a2-selective targets. Notably, these extracellular loop regions directly contact the exocytoplasmic domain of CDC50A (residues 205–217; UniProt ID: Q8VEK0), which is essential for forming a stable and functionally active Atp8a2-CDC50A complex, as demonstrated by domain-swap chimera experiments [20]. Thus, targeting these specific low-conservation loops with a CDC50A-derived peptide not only leverages their functional importance but also ensures preferential specificity and reduced cross-reactivity with Atp8a1. The proposed CDC50A-derived peptide (Q8VEK0 205-217; GIAWWTDKNVKFR) is predicted to bind Atp8a2 externally and disrupt its interaction with CDC50A. Given evidence of CDC50A-Atp8a2 interaction in the endoplasmic reticulum [78], we additionally incorporated the TAT peptide (GRKKRRQRRRPQ), a cell-penetrating peptide (CPP) derived from human immunodeficiency virus, to facilitate intracellular delivery. TAT was chosen for its established utility in molecular biology and therapeutics, particularly for delivering drugs and peptides [79]. To enable sufficient movement of both TAT and the effector peptide so that they could exert their biological activities, we additionally inserted a flexible GAG linker, generating the PI8a2 peptide (GRKKRRQRRRPQ-GAG-GIAWWTDKNVKFR). Additionally, as a specificity control, we created a scrambled peptide by modifying key amino acids considered to be responsible for electrostatic interactions with the partner molecule, resulting in the control scr-PI8a2 peptide (GRKKRRQRRRPQ-GAG-GIAKWTRKAVEFD). All molecular visualization were produced using the ChimeraX platform [67].

### Surface expression assay

For proteolysis experiments, neurons were incubated and lysed as described previously [42]. In brief, neurons were incubated with 0.25 mg/ml α-chymotrypsin (Sigma) in PBS for 5 min with 40 rpm agitation at 37 °C. After removal of enzymatic solution, cells were first washed for 1 min with an ice-cold solution containing 0.25 mg/ml trypsin-chymotrypsin inhibitor (Sigma) to inhibit further proteolysis. Next, plates were washed three times with ice-cold PBS before proceeding to harvesting.

### Transfection and morphometrics

DNA transfection was performed after a culturing period of 8 DIV with the specified construct using Lipofectamine 2000 (Invitrogen). For the morphometric analyses, fluorescence images were acquired using a confocal laser-scanning microscope (Leica Sp8) with a 63x oil immersion objective. For analysis of dendrites, all images were obtained with sequential acquisition settings and 1024 × 1024 pixel resolution. Each image was a z-series projection of images taken at 1-μm depth intervals for dendrites.

For morphometric analyses, neurons were analyzed on DIV 14. Total dendritic length and complexity were calculated using Fiji as described [15]. Briefly, a z-stack acquisition was imported, calibrated, and traced manually using the Simple Neurite Tracer plugin. Total dendritic length was then computed. For three-dimensional Sholl analysis, the shell interval was set to 5 μm using a plugin available for Fiji.

For time-lapse neurite length analysis, cells were imaged using an Incucyte® S3 Live-Cell Analysis System (Sartorius). Cultures were placed in the machine either on DIV 9 and continuously treated with scr-PI8a2 / PI8a2 for 3 days, or on DIV 10 for 5.5 days. Images were acquired every 6 h using a 20× objective in either the red channel (excitation: 567–607 nm; emission: 622–704 nm; exposure time: 400 ms) or green channel (excitation: 441–481 nm; emission: 502–544 nm; exposure time: 300 ms). A total of 16 images (875 × 645 µm/image) were obtained per well of a size of 1.9 cm², containing ca. 0.18 × 10^6^ cells. The total neurite length for each well was analyzed using the Incucyte® Neurotrack Analysis Software Module (Sartorius) as previously described [10]. The total neurite length was always normalized per well to the first timepoint after treatment start.

### Immunocytochemistry

Hippocampal neurons were fixed with 4% paraformaldehyde and 4% sucrose in PBS (pH 7.4) at room temperature for 20 min. Antibodies were diluted in GDB (0.1% gelatin, 0.3% Triton X-100, 15 mM Na_2_HPO_4_, 400 mM NaCl), and cells were incubated overnight with primary antibodies at 4 °C for 45 min with secondary antibodies at room temperature. Hoechst staining (1:6000 in 1× PBS; Serva Electrophoresis) was used for visualization of nuclei. Coverslips were mounted with Mowiol 488 (Calbiochem).

Proximity ligation assay was performed using the Duolink ® In Situ Red Starter Kit (Mouse/Rabbit; Sigma-Aldrich) following the manufacturer´s instructions. Protein-protein interactions were detected using primary antibodies raised in different species, followed by species-specific secondary antibodies conjugated to complementary oligonucleotides. Upon close proximity of the target proteins, oligonucleotides were ligated and amplified by rolling circle amplification, generating a fluorescent signal detectable by fluorescence microscopy.

The following primary antibodies were used: mouse anti-CDC50A (1:500, Merck Millipore) and rabbit anti-Atp8a2 (1:1000, Origene).

### Immunohistochemistry

For immunohistochemistry, mice were euthanized 21 days after rAAV-injection or 7 days following NMDA injection by intraperitoneal injection with 400 mg/kg BW pentobarbital (Narcoren; Merial). Following euthanasia, transcardial perfusion was performed using ice-cold 1× PBS and 10% formalin. Eyes were enucleated and post-fixed for 20 min. To dissect the retina, the cornea, iris, lens, and vitreous body were removed under a stereomicroscope. For whole-mount retinal immunostaining, the retina was isolated from the sclera and pigment epithelium and transferred into PBS. Optic nerves were cryoprotected in anti-freeze solution at −20 °C before being embedded in Tissue-Tek and cryosectioned. Optic nerve sections of 10 μm thickness were obtained with a Leica CM1950 cryostat (Leica) and mounted on Superfrost Plus slides (Thermo Scientific). Slides were stored at −20 °C until analysis. Optic nerves were immunostained directly on microscope slides. Slides were blocked in 0.5% Triton X-100 and 10% fetal calf serum (FCS) in 1× PBS for 1.5 h at room temperature. Free-floating immunostaining was performed for retinal whole mounts using blocking solution (1% Triton X-100, 10% FCS in 1× PBS) overnight at 4 °C. The following primary antibodies were diluted in respective blocking solution and incubated overnight at 4 °C: mouse anti-Brn3a (1:500; Santa Cruz Biotechnology), rabbit anti-Tau (1:1000; Synaptic Systems), mouse anti-non-phosphorylated Neurofilament H (NF-H) (SMI-32, 1:750; BioLegend). The following secondary antibodies were diluted in blocking solution and incubated for 90 min at room temperature: goat anti-mouse IgG (H+L) Alexa Fluor 488 (1:1000; Life Technologies), goat anti-rabbit IgG (H+L) Alexa Fluor 594 (1:1500; Life Technologies). Nuclei were labeled with Hoechst (1:6000 in 1× PBS; Serva Electrophoresis) for 5 min. Images were acquired at a Leica Sp8 confocal laser scanning microscope with a 40× objective and 0.75× zoom.

### Quantification of RGCs and axonal damage

For quantification of RGCs in whole-mounted retinas, Brn3a-positive cells were manually counted in eight images per retina, evenly sampled from both central and peripheral regions, using ImageJ/Fiji as described [29, 30]. Total RGC counts were summed for each eye. Relative RGC numbers were calculated by normalizing counts from shAtp8a2- or shAtp8a1-injected eyes to shControl-injected eyes, or by normalizing NMDA-treated eyes to PBS-treated controls in the same mouse. Five mice were analyzed per condition. For axonal damage analysis, the number of SMI-32-positive clusters was quantified in three to four sagittal optic nerve sections (10 µm thick) using ImageJ/Fiji and normalized to the analyzed area [30]. Four mice were analyzed per condition.

### Assessment of cell death

Hippocampal neurons were transfected on DIV 8 and treated with NMDA for 10 min on DIV 9, or treated with peptide PI8a2 or control peptide (scr-PI8a2) at DIV 10 for 24 or 48 h. 24 h after NMDA stimulation or the end of the peptide treatment, neurons were fixed with 4% paraformaldehyde and 4% sucrose in PBS (pH 7.4) at room temperature for 20 min. Nuclei were counterstained with Hoechst (1:6000 in 1x PBS; Serva Electrophoresis). All transfected cells per coverslip were analyzed, and the percentage of dead cells was determined by observing the nuclei shape. Two to three coverslips were quantified per condition.

### Gene ontology enrichment analysis

Protein identifications from published ATP8A2 immunoprecipitation-mass spectrometry datasets derived from HEK293T cells and bovine retinal tissue were obtained from ref. [47]. Protein entries were converted to gene symbols, redundant entries were consolidated, and manually curated contaminant proteins were removed prior to analysis. Expanded co-purifying protein sets were generated from the top ~200 ranked proteins of each dataset based on the provided identification confidence scores (log(prob)), resulting in datasets comprising 175 genes for HEK293T cells and 167 genes for retinal tissue. Gene Ontology (GO) Molecular Function enrichment analysis was performed using ShinyGO version 0.85.1 with *Homo sapiens* selected as the reference organism and an FDR threshold of 0.05. The top 25 enriched pathways were used for downstream analysis and visualization. Cytoskeleton-associated proteins identified within significant GO-enriched pathways were further analyzed using STRING to generate protein–protein interaction networks. Percentages indicate the proportion of cytoskeleton-associated proteins relative to the total number of proteins included in the corresponding GO enrichment analysis.

### Biochemical fractionation

PSDs were isolated from hippocampi of 6-week-old Sprague–Dawley rats as previously described [80]. Briefly, hippocampi from five animals were rapidly dissected immediately after sacrifice, while tissues dissected beyond this time window were discarded. Tissue was homogenized in four volumes of ice-cold 0.32 M sucrose buffer containing 1 mM HEPES, 1 mM MgCl₂, 1 mM NaHCO₃, 0.1 mM PMSF, and protease inhibitors (Complete; Boehringer Mannheim GmbH) using a Teflon–glass homogenizer (10 strokes, 700 rpm). The homogenate was centrifuged at 1000 × *g* for 10 min, and the resulting supernatant was further centrifuged at 3000 × *g* for 15 min to obtain a crude synaptosomal fraction. The pellet was resuspended in 0.32 M sucrose buffer and separated on a discontinuous sucrose gradient (0.85–1.0–1.2 M) by centrifugation at 82,500 × *g* for 2 h. The fraction collected between 1.0 and 1.2 M sucrose was diluted 1:1 with 1% Triton X-100 in sucrose buffer and gently stirred for 15 min at 4 °C before centrifugation at 82,500 = × g for 30 min. The resulting pellet was subjected to a second sucrose gradient (1.0–1.5–2.1 M) and centrifuged at 100,000 × *g* for 2 h at 4 °C. The fraction between 1.5 and 2.1 M sucrose was collected, diluted with an equal volume of 1% Triton X-100 containing 150 mM KCl, and centrifuged at 100,000 × *g* for 30 min at 4 °C to obtain purified PSDs, which were stored at −80 °C until use.

### Antibodies

The following antibodies were used: mouse anti-tubulin (T9026, Sigma); rabbit anti-cFos (sc-52, Santa Cruz Biotechnology); mouse anti-NeuN (MAB377, Merck Millipore); rabbit anti-Atp8a2 (sc-292155, Santa Cruz); rabbit anti-Atp8a2 (AP50308PU-N, Origene); mouse anti-caspase 3 (sc-7272, Santa Cruz Biotechnology); rabbit anti-cleaved caspase 3 (9661, Cell Signaling); mouse anti-CDC50A (MABN2616, Merck Millipore); mouse anti-Non-phosphorylated Neurofilament H (NF-H) (SMI-32; 801701, BioLegend); rabbit anti-Tau (314002, Synaptic Systems); mouse anti-Brn3a (sc-8429, Santa Cruz Biotechnology); mouse anti-PSD-95 (192-757, Abcam); rabbit anti-Synaptophysin (32-127, Abcam); rabbit anti-vGlut1 (75066, Abcam), rabbit anti-Atp8a1 (21565-1-AP, Proteintech). Rabbit anti-Atp8a1 was kindly provided by Dr. Xiao-Song Xie. The following secondary antibodies were used goat anti-mouse IgG (H+L) peroxidase (115-035-003, AffiniPure Jackson Immuno Research), goat anti-rabbit IgG (H+L) peroxidase (111-035-144, AffiniPure Jackson Immuno Research); goat anti-mouse IgG (H+L) Alexa Fluor 488 (A11001, Life Technologies), goat anti-mouse IgG (H+L) Alexa Fluor 594 (A11005, Life Technologies), and goat-anti-rabbit IgG (H+L) Alexa Fluor 488 (A11008, Life Technologies).

### Statistics and data analysis

All plotted data represent mean ± SEM. Statistical details such as *N* numbers, *p*-values and tests performed are available in the respective figure legends and in the source data file. All data were tested for normality. Parametric tests were applied when normality was confirmed; non-parametric equivalents were used otherwise. Results were considered to be statistically significant for significance levels of *p* < 0.05 (*), *p* < 0.01 (**), *p* < 0.001 (***), or *p* < 0.0001 (****). For animal studies, mice were randomly assigned to experimental groups. For in vitro experiments, culture conditions were distributed across wells to avoid positional bias on the plate or plates were randomly assigned to conditions. Utilized animals were male mice between 2 and 3 months of age, unless otherwise specified. Sample sizes were not determined by formal a priori power analysis, as the majority of experiments were exploratory in nature and no reliable estimates of effect size were available from prior literature for the specific experimental conditions used. Instead, sample sizes were based on those routinely used in the field for primary neuronal culture experiments and for in vivo experiments; group sizes of 4–6 animals per condition were chosen based on previously published studies using the same models and were determined to be sufficient to detect biologically meaningful differences based on prior experience in the laboratory. Where possible, analyses were performed using automated software to eliminate observer bias: pSIVA fluorescence quantification and neurite length analyses were performed using Incucyte® automated analysis software, qRT-PCR data were quantified automatically by the StepOne software, and western blot densitometry was performed using ImageJ software, all without investigator input into individual measurements. For analyses requiring manual quantification, blinding was not always feasible (i.e., NMDA neurotoxicity is clearly distinctive). For transfection-based experiments, blinding was not possible as the fluorescent transfection marker was required for cell identification.

Supplementary Information including 4 supplementary figures and relative legends is available on the journal website. All data and statistical tests used to generate the graphs are provided in the data source file. Uncropped original blot images are provided in the accompanying original data file.

## Supplementary information

Supplemental Information

Original data_Uncropped blots

Original data_Source Data

## Data Availability

All data and statistical values are provided in an additional file (source data file). Additionally, uncropped blots are provided in a dedicated supplementary file.
