# Supplementary material for: Loss of Atp8a2 drives neurodegeneration through the dysregulation of spatiotemporal phosphatidylserine externalization in mature neurons
Source: Cell Death Dis. 2026 Jul 22;17(1):652. doi: 10.1038/s41419-026-09097-y (PMC13392023; doi:10.1038/s41419-026-09097-y)
Supplement: Supplementary file 1 — Supplemental Information [file 41419_2026_9097_MOESM1_ESM.pdf]

SUPPLEMENTAL INFORMATION

Loss of Atp8a2 drives neurodegeneration through the dysregulation of spatiotemporal phosphatidylserine externalization in mature neurons

Adriana Schneider[1], Alexandra B. Merkel[1], Nunzio Perta[2,3], Lisa Ruff[1], Netta Ussyshkin[1], Paula Zimmer[1], Bahar Aksan[1], Amandine Lepeuve[1,4], Laura D'Andrea[5], Silvia Pelucchi[5], Elena Marcello[5], Daniele Di Marino[2,3,§] and Daniela Mauceri [1,4*]

[1] Department of Neurobiology, Interdisciplinary Centre for Neurosciences (IZN), Heidelberg University, Germany

[2] Department of Life and Environmental Sciences, Marche Polytechnic University, Ancona, Italy

[3] Department of Neuroscience, Istituto di Ricerche Farmacologiche Mario Negri IRCCS, Milan, Italy

[4] Marburg University, Institute of Anatomy and Cell Biology, Dept. Molecular and Cellular Neuroscience, Germany

[5] Department of Pharmacological and Biomolecular Sciences “Rodolfo Paoletti”, Università degli Studi di Milano, Milan, Italy.

§ deceased

* To whom correspondence should be addressed.

Prof. Dr. Daniela Mauceri

E-mail: mauceri@uni-marburg.de; mauceri@nbio.uni-heidelberg.de

Marburg University, Institute of Anatomy and Cell Biology, Dept. Molecular and Cellular Neuroscience, Karl-von-Frisch-Straße 16, 35043, Marburg Germany;

Neurobiology, Heidelberg University, Im Neuenheimer Feld 366, 69120 Heidelberg Germany, +49 06221 5416508

This document contains Supplemental Figure 1, 2, 3, 4 and relative figure legends.

Supplemental Figure 1

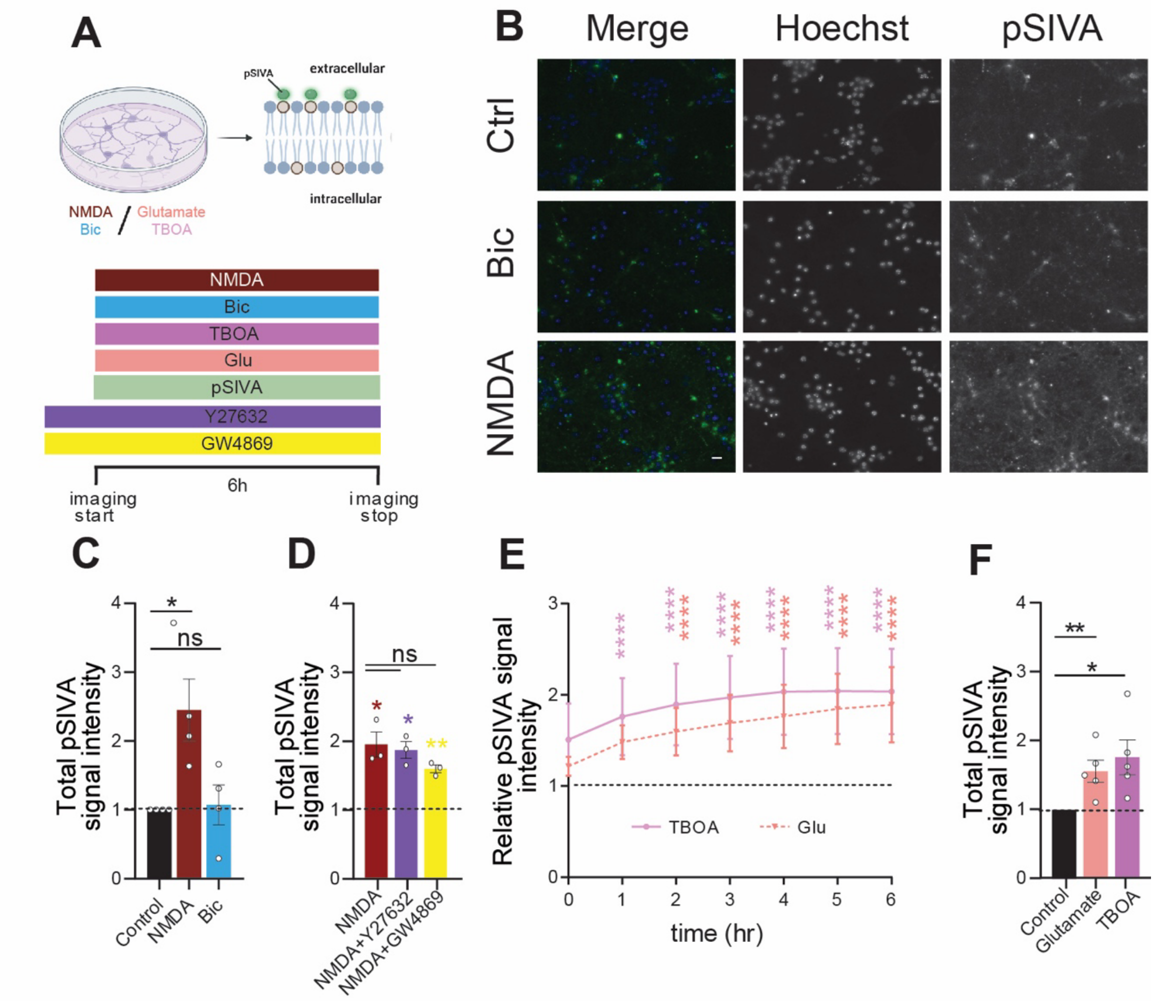

Supp. Fig. 1 PS is exposed after excitotoxity but not after increased synaptic activity

A Schema of pSIVA time-lapse imaging experiments **B** Representative pSIVA images of neurons treated with NMDA, Bic, or untreated 2h after treatments. Images acquired with TI-high content screening microscope. Scale bar = 20µm. **C** Integrated relative pSIVA signal intensity over time. Images acquired with TI-high content screening microscope. N= 3 independent cultures. **D**, Relative integrated pSIVA fluorescence over 4h in neurons treated with NMDA and pretreated for 30' with Y27632 or GW4869 and normalized to initial timepoint and untreated control. N = 3 independent cultures. **E, F** Relative pSIVA fluorescence intensity over time (E) and integrated (F), in neurons

treated with TBOA, glutamate, or untreated and normalized to initial timepoint and untreated control. N = 5 independent cultures. Two-way ANOVA with repeated measures with Dunnett's post hoc test (E), one-way ANOVA with Dunnett's post hoc test (C, D, F) or One sample t-test (D). Graphs represents mean ± SEM. Single values are represented as data points. ****$p < 0.0001$; **$p < 0.01$; *$p < 0.05$. ns non significant $p > 0.05$.

Supplemental Figure 2

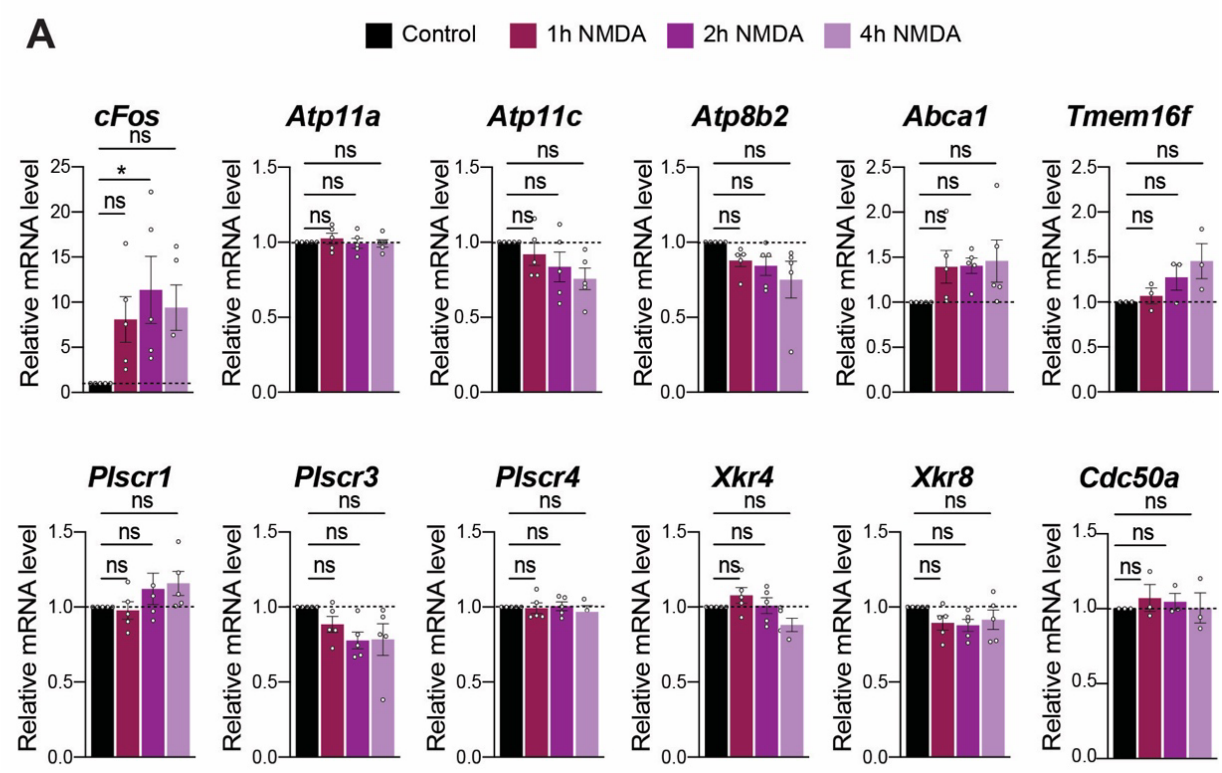

Supp. Fig. 2 Expression of PS shuttling enzymes after excitotoxity.

A QRT-PCR analysis of *cFos, Atp11a*, *Atp11c*, *Atp8b2*, *Abca1*, *Tmem16f, Plscr1*, *Plscr3*, *Plscr4*, *Xkr4*, *Xkr8, and Cdc50a* mRNA expression in hippocampal neurons ± NMDA treatment for 1, 2 or 4 h. Expression levels were normalized to *Gusb* and control. N = 3-5 independent cultures. One-way ANOVA with Dunnett's post hoc test. Graphs represent mean ± SEM. Single values are represented as data points. $^{*}p < 0.05$; ns non significant $p > 0.05$.

Supplemental Figure 3

A

	mATP8A2	mCDC50A
1	7vgj_A	7vgh_A
2	6roh_A	6lkn_J
3	6k7i_A	6k7j_C
4	6psy_A	7bsv_C
5	6k7k_A	7whw_B
6	6k7n_A	7oh5_C
7	6k7h_A	6roh_C
8	6k7g_A	6lcr_B
9	7vgh_B	7vgh_A
10	7py4_A	7bsv_C
11	7oh4_A	
12	7pem_A	
13	6k7l_A	
14	7vgi_B	
15	6psx_A	
16	7kyc_A	
17	6lcp_A	
18	7drx_A	
19	7whw_A	
20	7whv_A	

B

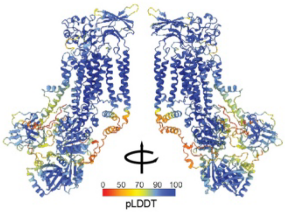

C

Organism	Protein	Name	UniProt ID
Mus musculus	ATP8A2	mmATP8A2	P98200
Mus musculus	ATP8A2	mmATP8A1	P70704
Homo sapiens	ATP8A2	hsATP8A2	Q9NTI2
Homo sapiens	ATP8A1	hsATP8A1	Q9Y2Q0-2
Bos taurus	ATP8A2	btATP8A2	C7EXK4
Homo sapiens	ATP9A	hsATP9A	O75110
Homo sapiens	ATP8B1	hsATP8B1	O43520
Homo sapiens	ATP10A	hsATP10A	O60312
Saccharomyces cerevisiae	Dnf1	scDnf1	P32660
Saccharomyces cerevisiae	Drs2	scDrs2	P39524
Saccharomyces cerevisiae	Dnf2	scDnf2	Q12675
Saccharomyces cerevisiae	Dnf3	scDnf3	Q12674
Saccharomyces cerevisiae	Neo1p	scNeo1	P40527

D

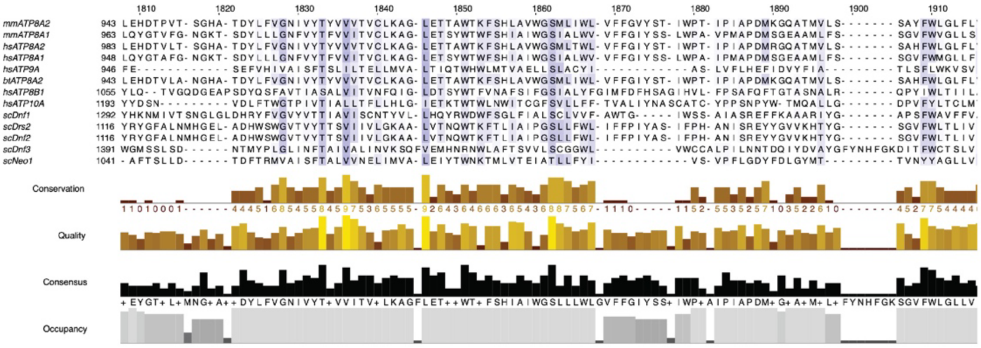

E

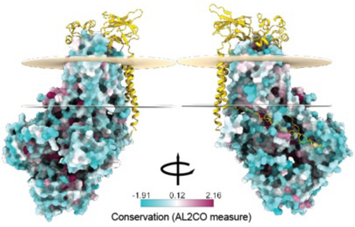

F

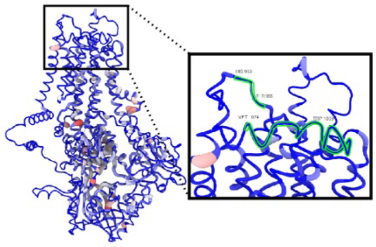

G

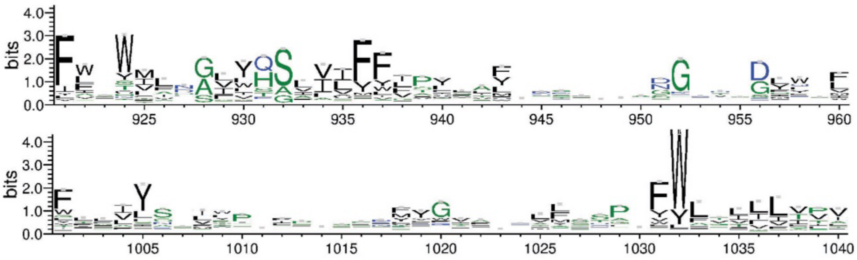

Supp. Fig. 3 Structural modelling and conservation mapping the mouse ATP8A2-CDC50A complex.

A List of PDB templates automatically selected by AlphaFold2-multimer (PDB100 library) for modelling mouse ATP8A2 (left column) and CDC50A (right column) protein structures. **B** Ribbon representation of the top-ranked *Mus musculus* ATP8A2-CDC50A complex predicted by AlphaFold2-multimer, color-coded by per-residue pLDDT confidence scores. **C** Sequences list used for the Multiple Sequence Alignment (MSA) of the ATP8A2 protein conducted with MUSCLE: organism, protein, common name, and UniProt accession. Inclusion of diverse vertebrate and yeast isoforms provides the phylogenetic depth required for reliable conservation estimates. **D** Multiple-sequence alignment (MSA) of a selected ATP8A2 region across orthologues and paralogues (eukaryotic flippases), generated with MUSCLE and visualized in Jalview. Divergent positions cluster at loops 953-955 and 1009-1024 of mouse ATP8A2, pinpointing the low-conservation areas chosen for selective peptide targeting. **E** Conservation map of ATP8A2 after membrane placement and energy-minimization. The molecular surface is coloured with AL2CO entropy values calculated from a multi-species eukaryotic flippases sequence alignment (scale bar: cyan = variable; magenta = highly conserved). CDC50A is represented as a yellow ribbon structure, emphasizing its overall topology and secondary structural elements. **F** ConservFold conservation analysis of ATP8A2 protein. The structure with entropy value for each amino acid is viewed using the *worms* cartoon features of ChimeraX, with red being high and blue being low entropy/conservation. The *fatness* reflects the values of the *bfactor* attribute. The inset zooms on the two low-conservation ATP8A2 loops (residues 953–955 and 1009–1024, green contoured) that contact CDC50A residues 205-217 (Fig. 5C). **G** WebLogo representation of conservation scores for the

regions of interest, generated using ConservFold. Small letter heights confirm high variability, supporting their suitability for isoform-specific targeting.

Supplemental Figure 4

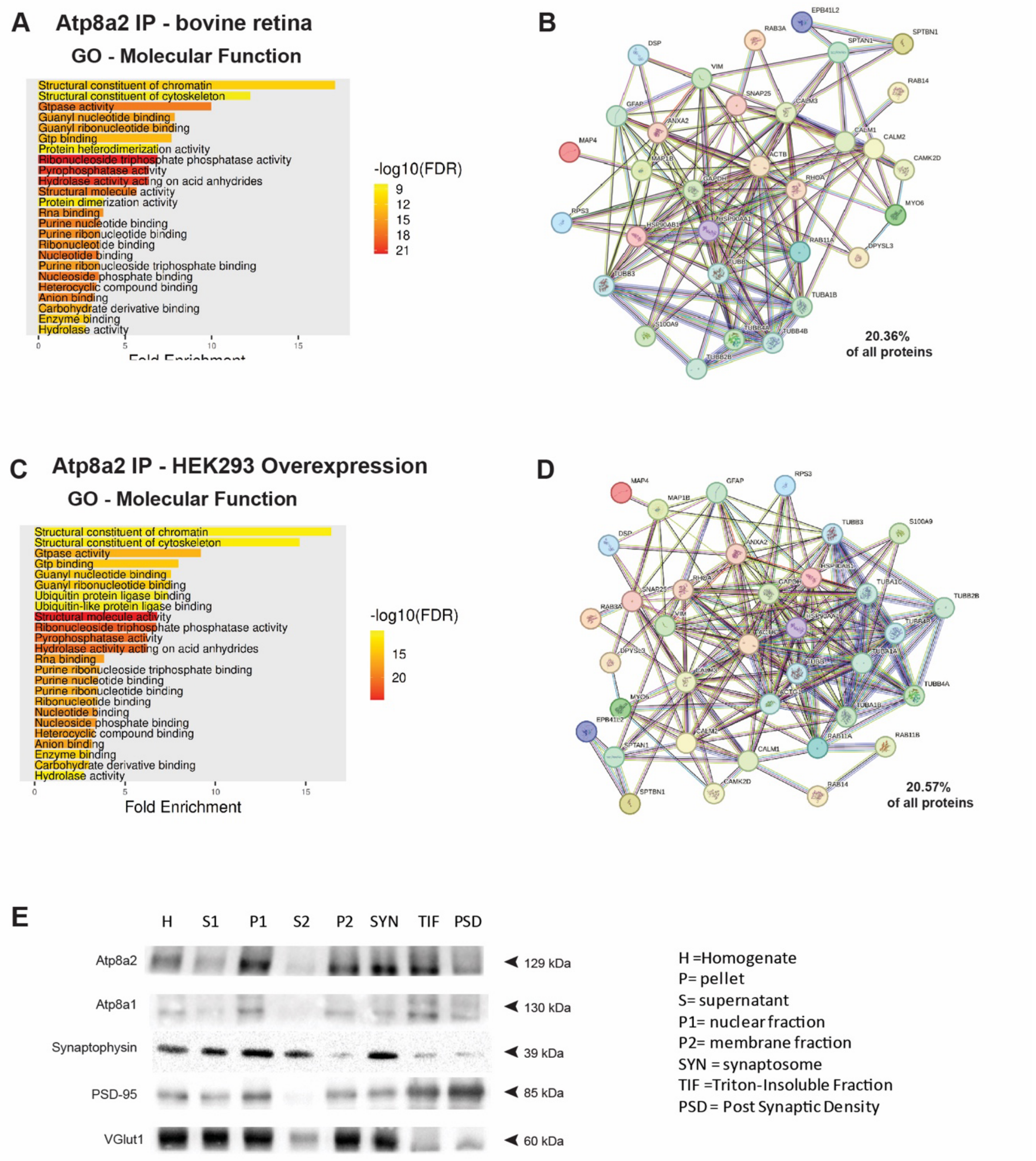

Supp. Fig. 4 Comparative pathway enrichment and network analysis, of Atp8a2- and Atp8a1-associated protein complexes and subcellular localization.

A,C Gene Ontology (GO) molecular function enrichment analyses performed on published Atp8a2-associated interactomic datasets derived from bovine retina (**A**) and HEK293T overexpression systems (**C**)(1). Bar plots display the top 25 enriched GO molecular function categories ranked by fold enrichment. Color scale indicates −log10(FDR). **B,D** STRING network analyses of proteins associated with cytoskeleton-related GO categories identified in the corresponding datasets shown in (**A**) and (**C**). Percentages indicate the proportion of displayed cytoskeleton-associated proteins relative to the total number of proteins included in the corresponding GO enrichment analysis. **E** Biochemical subcellular fractionation of hippocampal tissue showing distribution of Atp8a2 and Atp8a1 across homogenate (H), pellet (P), supernatant (S), nuclear fraction (P1), membrane fraction (P2), synaptosome (SYN), Triton-insoluble fraction (TIF), and postsynaptic density (PSD) fractions. Synaptophysin, PSD-95, and VGLUT1 were used as fraction-specific markers (pre or post-synaptic).

References cited in this document

1. Matsell E, Mazaheri M, Andersen JP, Molday RS. Structural and functional properties of the N- and C-terminal segments of the P4-ATPase phospholipid flippase ATP8A2. J Biol Chem. 2025;301(1):108065.
