# Supplementary figures and images for: Loss of Atp8a2 drives neurodegeneration through the dysregulation of spatiotemporal phosphatidylserine externalization in mature neurons
Source: Cell Death Dis. 2026 Jul 22;17(1):652. doi: 10.1038/s41419-026-09097-y (PMC13392023; doi:10.1038/s41419-026-09097-y)

Full uncropped Gels and Blots image(s)

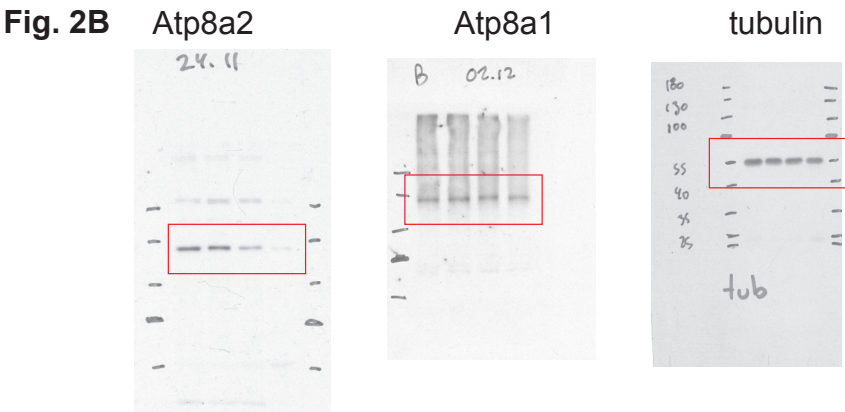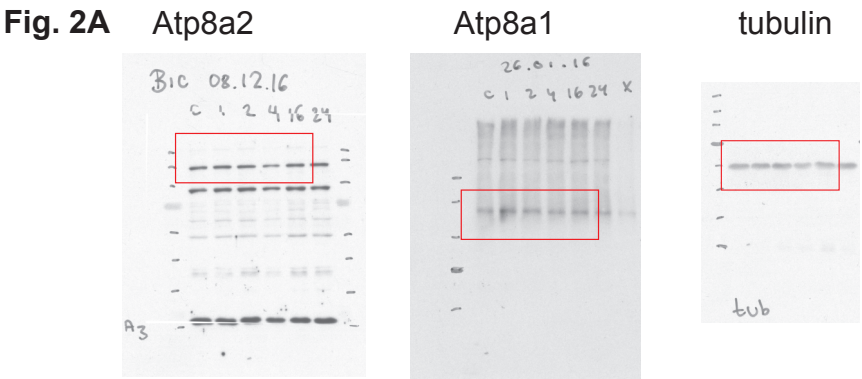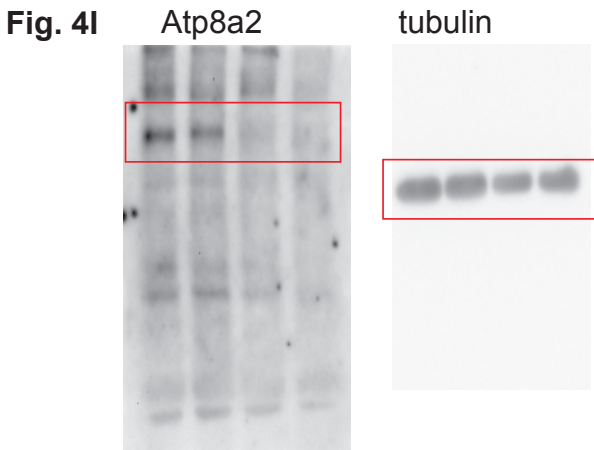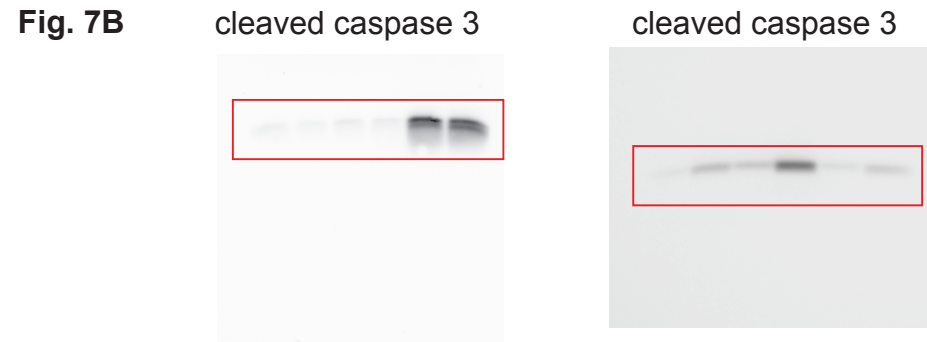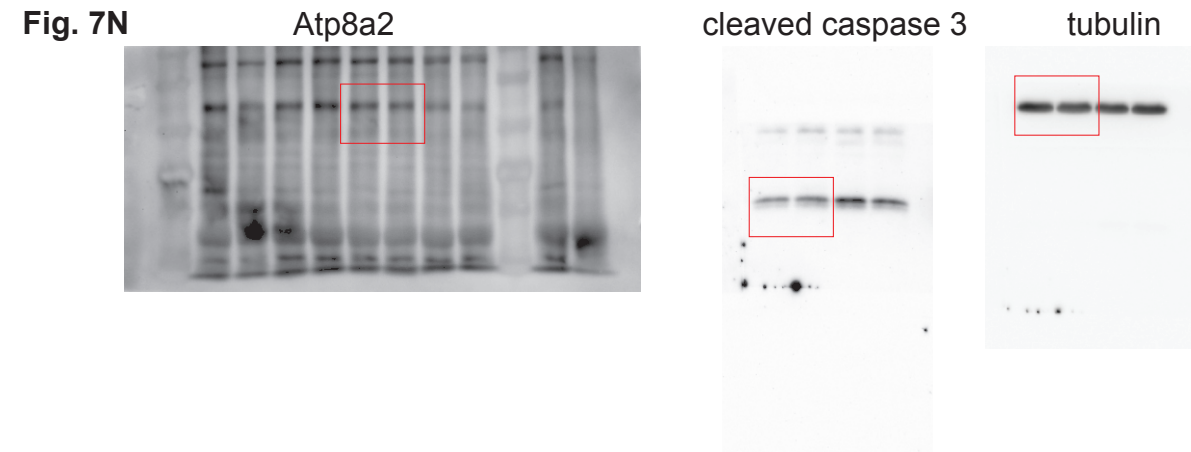

Supplemental Fig. 4E

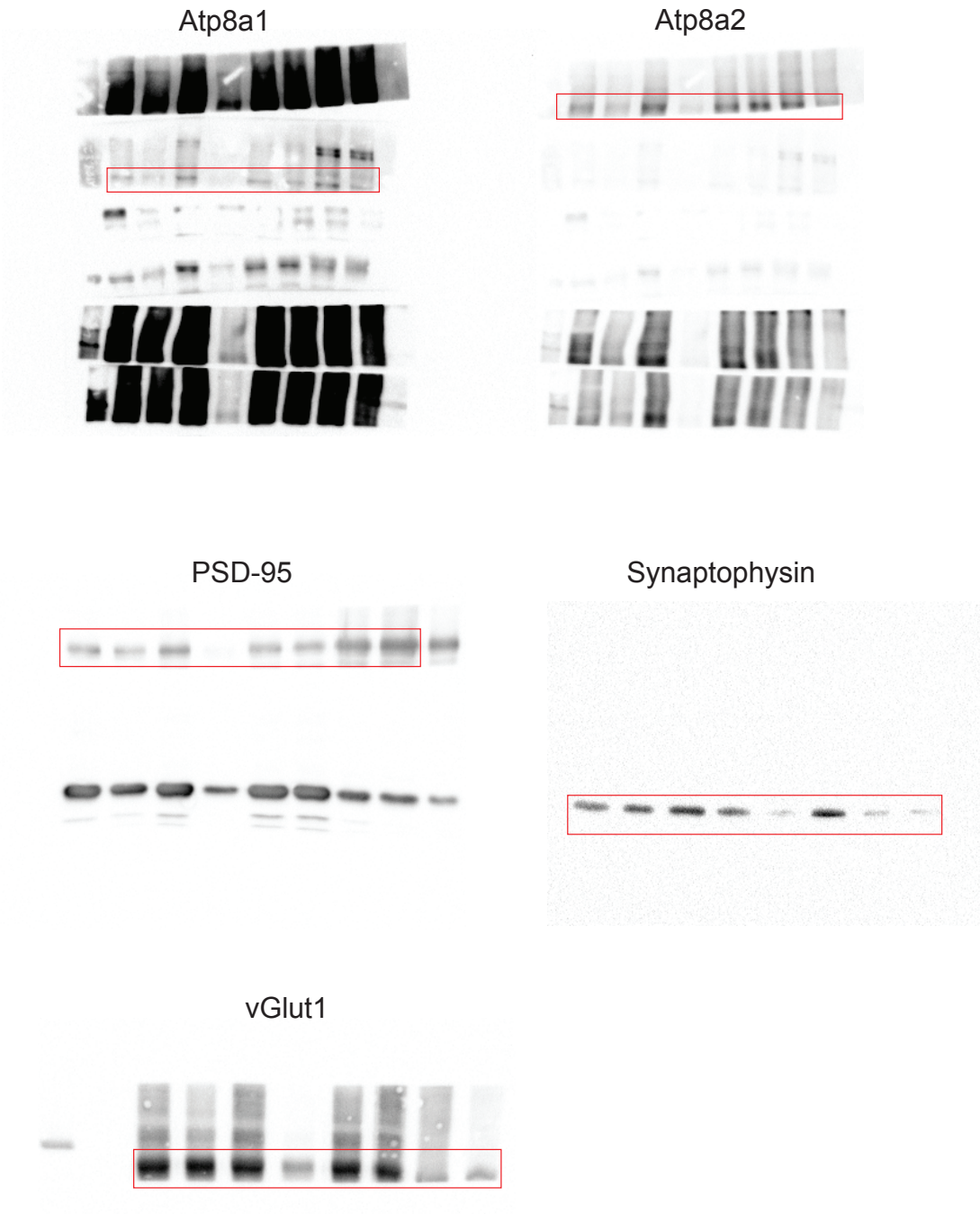

Supplement: Supplementary file 2 — Original data_Uncropped blots [file 41419_2026_9097_MOESM2_ESM.pdf]
